# Assessing the Potential of 1,2,3-Triazole-Dihydropyrimidinone Hybrids Against Cholinesterases: In Silico, In Vitro, and In Vivo Studies

**DOI:** 10.3390/ijms252011153

**Published:** 2024-10-17

**Authors:** Carlos M. Gastalho, Ana M. Sena, Óscar López, José G. Fernández-Bolaños, Alfonso T. García-Sosa, Florbela Pereira, Célia M. Antunes, Ana R. Costa, Anthony J. Burke, Elisabete P. Carreiro

**Affiliations:** 1 LAQV-REQUIMTE, Institute for Research and Advanced Training (IIFA), University of Évora, Rua Romão Ramalho, 59, 7000-671 Évora, Portugal; cgastalho@gmail.com (C.M.G.); ajburke@ff.uc.pt (A.J.B.); 2 Institute of Earth Sciences, Institute of Research and Advanced Training, University of Évora, 7000-671 Évora, Portugal; cmma@uevora.pt (C.M.A.); acrc@uevora.pt (A.R.C.); 3 Academic Clinical Center of Alentejo, C-TRAIL, Rua Romão Ramalho, 59, 7000-671 Évora, Portugal; 4 Department of Chemistry and Biochemistry, School of Sciences and Technologies, University of Évora, Rua Romão Ramalho, 59, 7000-671 Évora, Portugal; ana.m.sena98@gmail.com; 5 Departamento de Química Orgánica, Facultad de Química, Universidad de Sevilla, Apartado 1203, E-41071 Seville, Spain; osc-lopez@us.es (Ó.L.); bolanos@us.es (J.G.F.-B.); 6 Institute of Chemistry, University of Tartu, Ravila 14 A, 50411 Tartu, Estonia; alfonsog@ut.ee; 7 LAQV-REQUIMTE, Department of Chemistry, NOVA School of Science and Technology, Universidade Nova de Lisboa, 2829-516 Caparica, Portugal; florbela.pereira@fct.unl.pt; 8 Department of Medical and Health Sciences, School of Health and Human Development, University of Évora, Rua Romão Ramalho, 59, 7000-671 Évora, Portugal; 9 Faculty Pharmacy, University of Coimbra, Pólo das Ciências da Saúde, Azinhaga de Santa Comba, 3000-548 Coimbra, Portugal; 10 Departamento de Química, Coimbra Chemistry Centre-Institute of Molecular Sciences (CQC-IMS), University of Coimbra, 3004-535 Coimbra, Portugal; 11 Center for Neurosciences and Cellular Biology (CNC), Polo I, Universidade de Coimbra Rua Larga Faculdade de Medicina, Polo I, 1ºandar, 3004-504 Coimbra, Portugal

**Keywords:** 1,2,3-triazole, dihydropyrimidinone, antioxidant activity, cholinesterases, STD-NMR, docking

## Abstract

Combining the pharmacological properties of the 1,2,3-triazole and dihydropyrimidinone classes of compounds, two small families of mono- and di(1,2,3-triazole)-dihydropyrimidinone hybrids, A and B, were previously synthesized. The main objective of this work was to investigate the potential anti-Alzheimer effects of these hybrids. The inhibitory activities of cholinesterases (AChE and BuChE), antioxidant activity, and the inhibitory mechanism through in silico (molecular docking) and in solution (STD-NMR) experiments were evaluated. The 1,2,3-triazole-dihydropyrimidinone hybrids (A and B) showed moderate in vitro inhibitory activity on eqBuChE (IC_50_ values between 1 and 58.4 μM). The best inhibitor was the hybrid B4, featuring two 1,2,3-triazole cores, which exhibited stronger inhibition than galantamine, with an IC_50_ of 1 ± 0.1 μM for eqBuChE, through a mixed inhibition mechanism. Among the hybrids A, the most promising inhibitor was A1, exhibiting an IC_50_ of 12 ± 2 µM, similar to that of galantamine. Molecular docking and STD-NMR experiments revealed the key binding interactions of these promising inhibitors with BuChE. Hybrids A and B did not display *Artemia salina* toxicity below 100 μM.

## 1. Introduction

Alzheimer’s disease (AD) is known as a neurodegenerative disorder often characterized both by abnormal activities and intellectual impairment. Considered one of the main public health conditions, it currently affects more than 55 million people worldwide, particularly the elderly in low-and middle-income countries (WHO, 2023). It is predicted that, over the years, more people will suffer from the AD condition at some point and the population’s average age will also increase [1].

The pathophysiology of AD remains an enigma and without a cure, despite all the efforts of researchers in recent decades. AD is a multifactorial disease, and its causes could be associated with oxidative stress, metal ion dyshomeostasis, mitochondrial dysfunction, Aβ aggregation, hyperphosphorylation of Tau, and cholinergic dysfunction [2]. Oxidative stress and cholinergic dysfunction stand as central pillars in the pathogenesis of Alzheimer’s disease, profoundly influencing its onset and progression. The imbalance of oxidative stress is implicated in the onset of Alzheimer’s disease. Normally, the brain produces free radicals, which are regulated by antioxidative mechanisms. However, under pathological conditions, this balance is disrupted, leading to an overproduction of oxidizing species, including alterations in the balance of iron (Fe) and copper (Cu) [3,4].

The cholinergic mechanism involves the decline of the neurotransmitter acetylcholine (ACh) due to its hydrolysis by cholinesterases (ChEs), namely, acetylcholinesterase (AChE) and butyrylcholinesterase (BuChE); this process plays a direct role in cognitive deterioration. Additionally, it has been found that amyloid protein plaques are produced in the ACh deficiency state and that these can be reduced through the use of ChE inhibitors [5]. Neurotransmitter ACh can be hydrolyzed by both ChEs, even though the AChE is mostly neural in origin, while BuChE is mostly glial. ACh is preferentially catalyzed by AChE rather than BuChE [6]. Cholinesterase inhibitors (ChEIs) are considered the main symptomatic treatment for AD. For example, Donepezil, Galantamine, and Rivastigmine approved by the FDA [6] are ChEIs, which, in clinical practice, are often coupled with other molecules with a different mechanism of action (i.e., Memantine, Lecanemab, Aducanumab) for outcome improving.

A large number of heterocyclic compounds are currently available that feature the 1,2,3-triazole and the dihydropyrimidinone rings (DHPM), both pharmaceutically relevant, displaying a variety of biological, antitumor, antiviral, and anti-inflammatory activities and anti-Alzheimer’s, amongst others [7,8,9,10,11,12]. Pairing these two different rings in a single molecule enables it to target multiple molecular pathways simultaneously. The heterocyclic 1,2,3-triazole possesses robustness and resistance to hydrolysis, reduction, and oxidation, allowing it to serve as both a hydrogen bond donor and acceptor. This versatility has garnered significant interest among scientific researchers. Their growing use in medical fields is due to their stability and low toxicity. Indeed, they exhibit enhanced water solubility compared to many aromatic compounds and demonstrate greater stability in biological systems, making them particularly promising for drug development [9].

**Figure 1:**
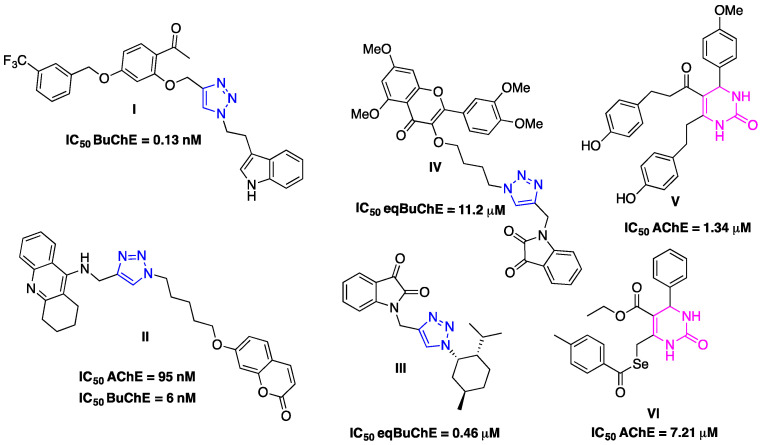
Compounds with anticholinesterase activity containing the 1,2,3-triazole (**I**–**IV**) and DHPM (**V**,**VI**) rings.

DHPMs are a major class of heterocyclic compounds, considering the different pharmacotherapeutic properties of these structures and their derivatives, with relevant anti-inflammatory, anti-hypertensive, antibacterial, antiviral, and anticancer properties [10]. In the literature, there are several examples of compounds containing the 1,2,3-triazole (I–IV) and DHPM (V and VI) rings in their structures, which exhibit anticholinesterase activity (Figure 1).

**Figure 2:**
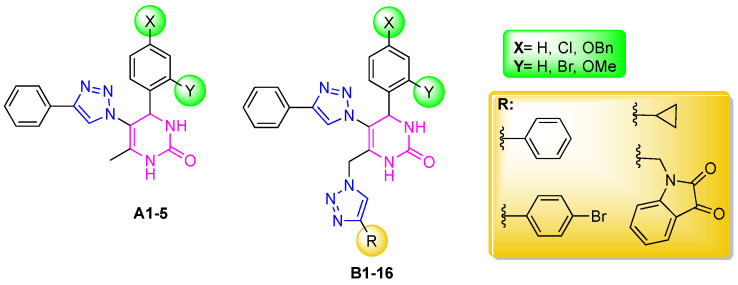
Structures of hybrids **A** and **B** [19].

Hybrids comprising the 1,2,3-triazole ring linked to at least one heterocyclic ring exhibit high biological activity against AD [8,12]. In Figure 1, the compounds feature the 1,2,3-triazole ring linked to various heterocyclics: paenol (I) [13], coumarin and tacrine (II) [14], isatin (III) [15], and quercetin (IV) [16]. This linkage is pivotal in enhancing their biological activity owing to their structural characteristics. Conversely, DHPMs have received limited attention for their anticholinesterase activity, with few examples reported in the literature. Figure 2 depicts DHPM derivatives (V) [17] and (VI) [18], wherein the DHPM ring is functionalized with aromatic substituents (V) and selenium element (VI). Although several 1,2,3-triazole-DHPM hybrids have been reported in the literature, none have been evaluated for their anticholinesterase activity [19].

Carreiro et al. synthesized two types of hybrids: the mono- and di(1,4-disubstituted-1,2,3-triazole)-DHPM, hybrids **A** and **B**, respectively (Figure 2) [19]. With the aim of creating more potent inhibitors, these hybrids were designated based on their structural characteristics, i.e., polarity, rigidity, ability to establish hydrogen bonds, and π–π interactions with a wide range of molecular targets.

The hybrids **A1**–**5** contain the 1,2,3-triazole unit at the C-5 position of the DHPM ring and the other hybrids **B1**–**16** contain two 1,2,3-triazole rings linked at the C-5 and C-6 positions of the methyl group of DHPM [19]. These new hybrids **A** and **B** were evaluated for their anticancer activity in vitro against six cancer cell lines: A549 and SW1573 (non-small cell lung), HBL-100 and T-47D (breast), HeLa (cervix) and WiDr (colon), and some of them have shown promising anticancer activity and therefore can be considered possible candidates as chemotherapeutic agents [19].

The aim of this work was to evaluate the cholinesterase inhibitory properties of hybrids **A1**–**3** and **B1**–**5**, as well as their antioxidant activity using the DPPH, ABTS, and FRAP colorimetric methods. Additionally, we investigated their toxicity. To obtain information on the inhibitory mechanism of the most promising hybrids, we conducted both in silico (Molecular Docking) and in solution (STD-NMR) studies.

## 2. Results and Discussion

### 2.1. Anticholinesterase Activity

**Figure 3:**
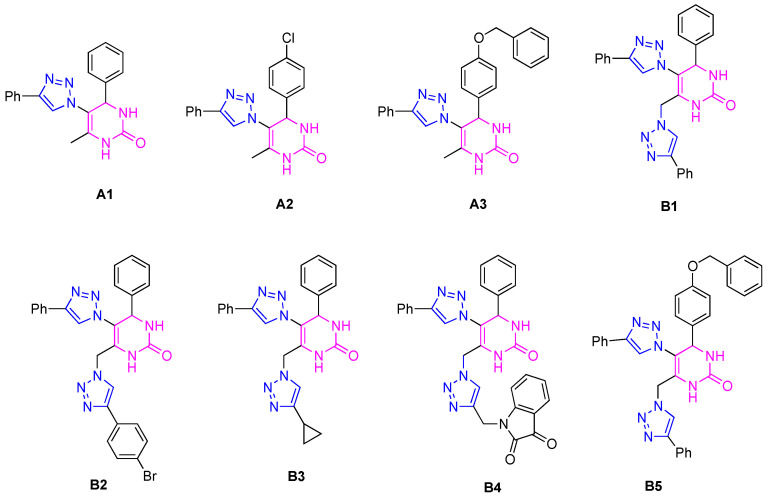
Structures of the hybrids evaluated in the cholinesterase inhibition assays.

In order to evaluate the inhibitory activity against cholinesterase enzymes (AChE and BuChE), well-established in vitro models were used—purified enzymes of animal origin, namely *electrophorus electricus* AChE (*ee*AChE) and equine serum BuChE (eqBuChE) [20,21,22,23,24,25,26]. Hybrids **A1**–**3** and **B1**–**5** (Figure 3) were evaluated in vitro for cholinesterase inhibition using Ellman’s colorimetric method [27]. Table 1 shows the results obtained for the 50% inhibitory concentration (IC_50_) of the studied hybrids against the enzymes *ee*AChE and eqBuChE. The values are expressed as the mean ± standard deviation (SD). By analyzing the results obtained for the IC_50_ (Table 1), it was found that none of the hybrids studied were effective inhibitors of *ee*AChE, as they presented IC_50_ levels higher than 100 µM.

Although the hybrids generally showed weak inhibition of eqBuChE, two compounds demonstrated significant activity: hybrid **A1** (with one 1,2,3-triazole ring) exhibited an IC_50_ = 12 µM, while hybrid **B4** (with two 1,2,3-triazole rings and a heterocyclic isatin) showed an IC_50_ = 1.0 µM. Additionally, three other compounds (**A2**, **B1,** and **B3**) exhibited moderate inhibition, with IC_50_ values ranging from 23 to 58 µM. Comparing the anticholinesterase activities of hybrids **A1** and **A2** with **B1** and **B3** suggests that hybrids **A** (with a single 1,2,3-triazole ring) are slightly more active than hybrids **B** (with two 1,2,3-triazole rings), with the exception of **B4** (see below). Nonetheless, hybrid **B4**, featuring two 1,2,3-triazole rings, emerged as the most potent eqBuChE inhibitor investigated in this study. Moreover, despite possessing two 1,2,3-triazole rings in its structure, it also has an isatin unit (with renowned biological activity), which is probably responsible for the high inhibition activity achieved. Hybrid **B4** was a better inhibitor than galantamine (IC_50_ = 10 µM).

**Figure 4:**
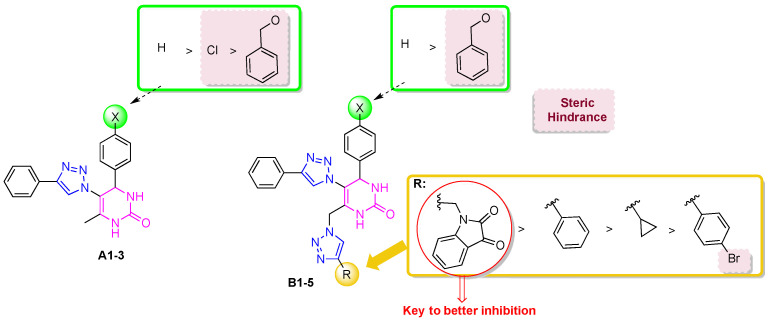
Structure−activity relationship of hybrids **A1**–**3** and **B1**–**5** in eqBuChE inhibition.

In the case of the **A1-3** hybrids, IC_50_ values were determined to be 12 ± 2 (**A1**), 24 ± 1 (**A2**), and >100 (**A3**) µM, the structure–activity relationship suggested that the unsubstituted phenyl ring in the 4-position of the DHPM core was the most active. However, the presence of the chlorine or benzyloxy groups in the para-position of the phenyl ring decreased the activity of the compounds, possibly due to the steric hindrance promoted by these groups. On the other hand, these groups also impart hydrophobicity to the hybrids, which could potentially interfere with their binding to the enzyme (Figure 4). In addition, hybrid **A1** showed the best inhibitory activity, with an IC_50_ value very similar to that of galantamine (IC_50_ = 10 µM).

**Table 1:** IC_50_ values for hybrids **A1**–**3** e **B1**–**5** against cholinesterases (*ee*AChE and eqBuChE).

IC_50_ ± SD (µM) ^a^		
Compound	*ee*AChE	eqBuChE
**A1**	>100	**12 ± 2**
**A2**	>100	24 ± 1
**A3**	>100	>100
**B1**	>100	53 ± 4
**B2**	>100	>100
**B3**	>100	58 ± 3
**B4**	>100	**1 ± 0.1** **Mixed inhibition ^b^** ***K*_ia_ = 1.1 ± 0.3 µM** ***K*_ib_ = 1.4 ± 0.2 µM**
**B5**	>100	>100
**Galantamine**	2.7 ± 0.2	10.0

^a^ [S] = 121 µM for *ee*AChE, 112 µM for eqBuChE; ^b^ methods of Dixon and Cornish-Bowden (Figure 5 and Figure 6) [28].

Regarding hybrids **B1**–**5**, these furnished IC_50_ values of 1 ± 0.1 (**B4**), 53 ± 4 (**B1**), 58 ± 3 (**B3**), >100 (**B2** and **B5**) µM. By comparing hybrids **B1**–**4,** the structure–activity relationship suggested that the best inhibitor is that which possesses an isatin moiety linked to the 1,2,3-triazole unit, while the phenyl and cyclopropane rings decreased the activity of the hybrids. Hybrids **B2** (with a 4-bromophenyl group linked to the 1,2,3-triazole) and **B5** (with a benzyloxy group in the para-position of the phenyl ring linked to the 4-position of the DHPM core) were the poorest inhibitors, possibly due to the presence of bulky groups in their structures, which could have compromised their binding to the enzyme’s active site. Notably, compound **B4** displayed very good anticholinesterase activity [15,29]. Furthermore, hybrid **B4** was a stronger inhibitor than galantamine by one order of magnitude.

**Figure 5:**
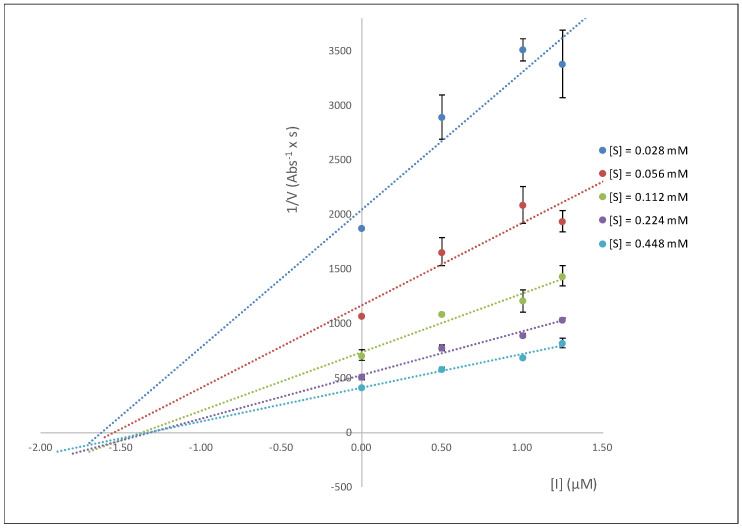
Graph 1/V vs. [I] study, where [I] is the **B4** inhibitor concentration.

**Figure 6:**
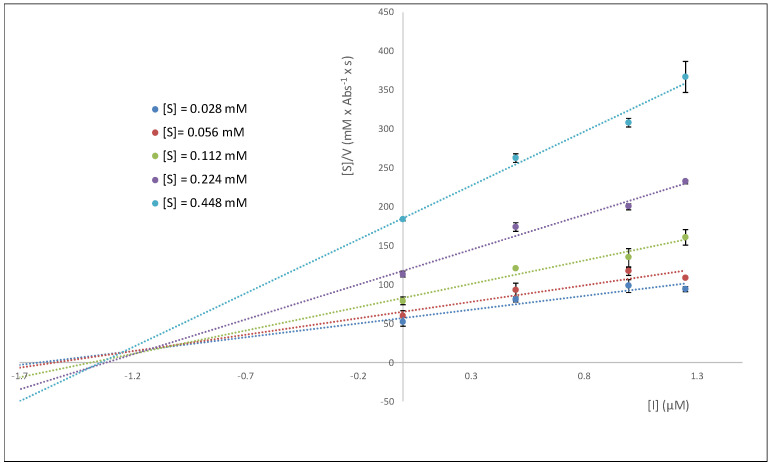
Graph [S]/V vs. [I] study, where [I] is the inhibitor **B4** concentration.

A study of the enzymatic kinetics for the inhibition of eqBuChE was carried out in order to determine the type of inhibition exhibited by the best inhibitor, notably, hybrid **B4**. The calculation of the kinetic parameters (non-linear regression, GraphPad 8.0) indicated a slight modification of K_M_ values and a decrease in V_max_ upon the increase in inhibitor concentration. Such observations are compatible with a mixed mode of inhibition, where the inhibitor binds the free enzyme and the E-S complex (K_ia_, K_ib_, respectively). This was further demonstrated using the Cornish-Bowden method [28], which considers two graphs: 1/V vs. [I] and [S]/V vs. [I], shown in Figure 5 and Figure 6, respectively. An analysis of the graphs reveals that a single intercept between the lines indicates the mixed mode of inhibition. The inhibition constants K_ia_ and K_ib_ were calculated, where K_ia_ represents the inhibition constant of the inhibitor binding to the enzyme-free complex (EF) and K_ib_ represents the inhibition constant of the inhibitor binding to the enzyme–substrate complex (ES). The value obtained for both constants was quite similar (K_ia_ = 1.1 µM, K_ib_ = 1.4 µM), confirming that the inhibitor binds both the free enzyme and the E-S complex, with analogous potency, leading to a change in the enzyme’s structure, altering the configuration of its active center, and thus preventing any enzyme activity (see below for a further discussion on this in the context of the docking and NMR studies).

### 2.2. In Silico Studies

The binding of the most promising inhibitors, **A1** and **B4**, to ChEs was explored and predicted through molecular docking using two software programs, Glide XP and AutoDock Vina. This study utilized several human protein X-ray crystal structures: AChE (PDB IDs 6O4W and 4EY7, both co-crystallized with donepezil) and BuChE (PDB IDs 4AQD, 7Q1M, and 4BDS, co-crystallized with β-alanine, a hydroxypropyl derivative, and tacrine, respectively). The calculated binding affinities obtained through molecular docking of the most promising ChE inhibitors identified are presented in Table 2. As shown in Table 2 using the Glide program, the known inhibitor donepezil exhibited a stronger predicted interaction with AChE compared to BuChE as expected. Conversely, **B4** exhibited stronger predicted interactions with BuChE than with AChE across all experiments, and, in each case, **B4** was predicted to interact more strongly than the co-crystallized ligands β-alanine (PDB ID 4AQD), the hydroxypropyl derivative, HPD, (PDB ID 7Q1M), and tacrine (PDB ID 4BDS).

**Figure 7:**
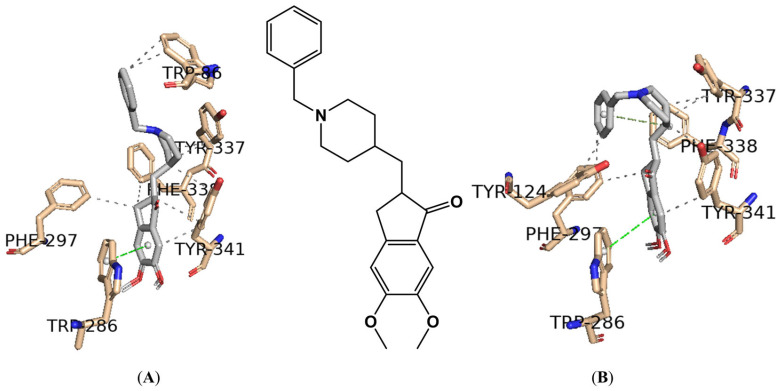
Interaction profile of the best-docked pose for the positive control, donepezil, against the AChE enzyme using (**A**) PDB ID 6O4W and (**B**) PDB ID 4EY7. Hydrophobic interactions are shown as black dashed lines while π-stacking interactions are depicted as green (parallel) and gray (perpendicular) dashed lines.

As observed experimentally, galantamine interacts with both AChE and BuChE simultaneously; however, gratifyingly, its interaction is predicted to be weaker than that of the **B4** derivative against BuChE. As shown in Figure 7, the best-docked pose for the positive control, donepezil, on the AChE enzyme was achieved using PDB IDs 6O4W (**A**) and 4EY7 (**B**).

The active site of human AChE has a long gorge approximately 20 Å in length (Figure 7, [30]), primarily composed of the catalytic active (or anionic) site (CAS) at the bottom of the gorge (His447, Ser203, Trp86, Tyr337) and the peripheral anionic site (PAS) near the entrance (His287, Ser293, Trp286, Tyr72). These two sites are connected by a narrow groove (Tyr124, Phe295, Tyr341). Compounds that can interact with both CAS and PAS are desirable due to their potential to exert multiple therapeutic effects [30,31], as exemplified by donepezil in Figure 7.

To validate the re-docking process, PyMOL software was used to superimpose the docked complexes of AChE (**A**) and (**B**) with donepezil onto the solved structures (PDB IDs 6O4W and 4EY7, respectively). The root-mean-square displacement (RMSD) between complex (**A**) and the solved structure was 0.161 Å, and between complex (**B**) and the solved structure was 0.225 Å, indicating high structural similarity in both cases.

**Figure 8:**
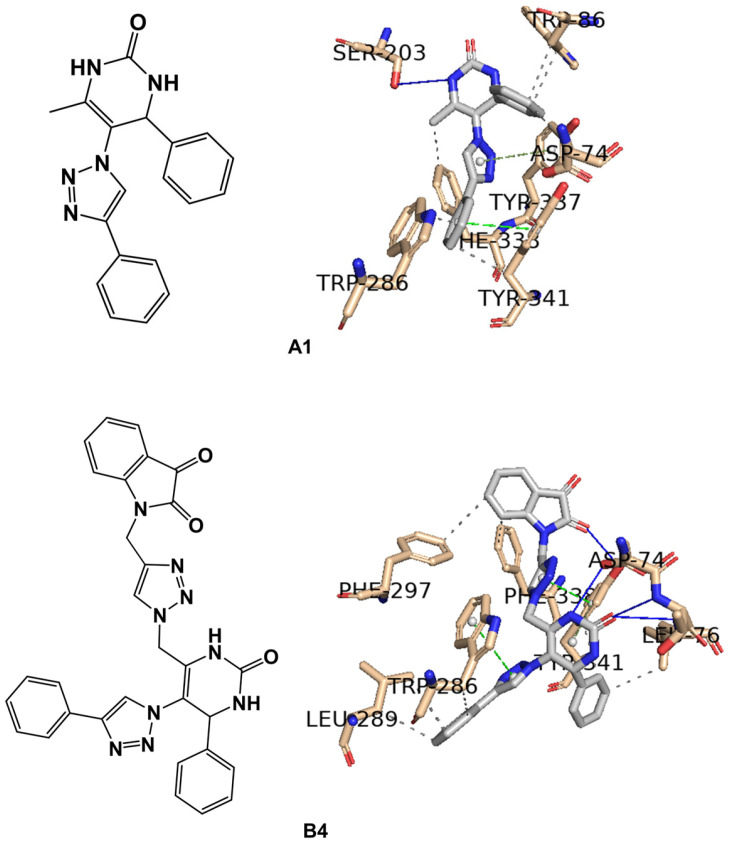
Interaction profiles of the best-docked poses for the **A1** and **B4** against the AChE enzyme. The hydrophobic interactions are shown as black dash lines and the π-stacking interactions are in green (parallel) and gray (perpendicular) dash lines. H-bond interactions are shown as blue continuous lines.

Figure 8 presents the best-docked poses for the two most promising AChE inhibitors, **A1** and **B4**, docked on PDB ID 6O4W.

Considering the calculation of the binding affinities for the hybrid **A1** against AChE using Glide and AutoDock Vina, which resulted in −3.72 kcal/mol and −11.75 kcal/mol, respectively, only the Glide docking score seems to justify the experimentally observed IC_50_ of >100 μM (Table 1 and Table 2). Moreover, the best-docked poses shown in Figure 8 for the hybrids **A1** and **B4** only seem to justify the lack of activity demonstrated against AChE for the hybrid **B4** (Table 1), as in this case, interactions are only observable with the PAS residue, specifically Trp286. In the case of hybrid **A1**, interactions are observable with the CAS residues (Trp86, Ser203, and Tyr337) as well as PAS (Trp286), as shown in Figure 8.

**Figure 9:**
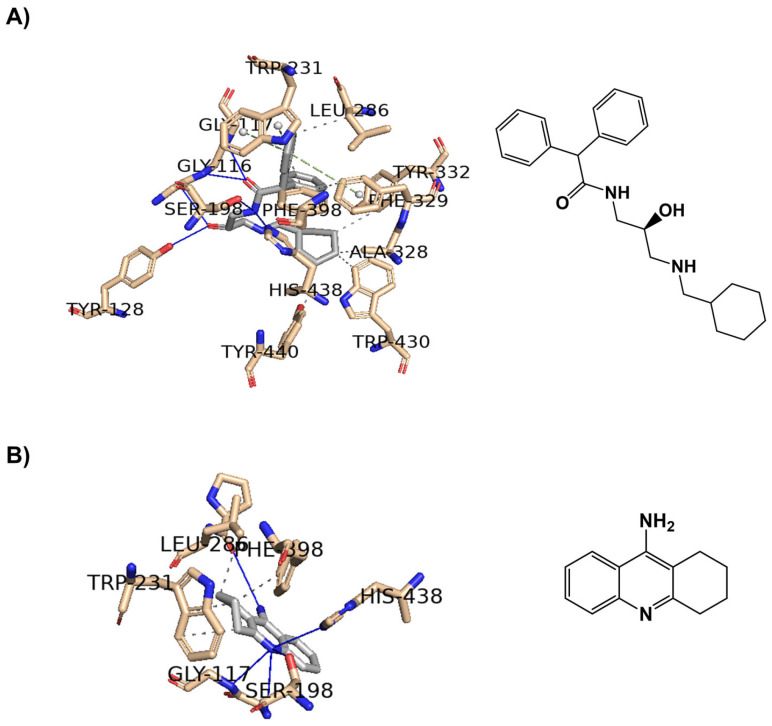
Interaction profiles of the best-docked poses for the two positive controls (**A**) 3-(cyclohexylmethyl)amino-2-hydroxypropyl (PDB ID 7Q1M) and (**B**) tacrine (PDB ID 4BDS) against BuChE enzyme. The hydrophobic interactions are shown as black dash lines and the π-stacking interactions are in green (parallel) and gray (perpendicular) dash lines. H-bond interactions are shown as blue continuous lines.

As with AChE, in BuChE, compounds capable of interacting with CAS residues (Trp82, Phe398, His438) and PAS residues (Asp70, Ser198, Phe329) appear to be more promising [31], as demonstrated for the hydroxypropyl and tacrine derivatives in Figure 9.

Similar to the approach used with AChE, the re-docking process was validated by using PyMOL software to superimpose the docked complexes of BuChE (**A**) and (**B**) with the hydroxypropyl derivative and tacrine onto the solved structures (PDB IDs 7Q1M and 4BDS, respectively). The root-mean-square deviation (RMSD) between complex (**A**) and the solved structure was 0.475 Å, and between complex (**B**) and the solved structure was 0.361 Å, indicating a high degree of structural similarity in both cases.

**Figure 10:**
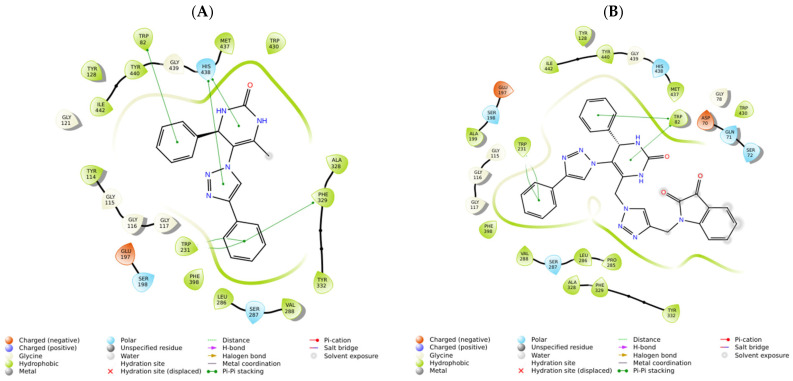
Interaction profiles of the best-docked poses for the (**A**) **A1** and (**B**) **B4** against BuChE enzyme (PDB ID 4AQD).

In the case of our stronger inhibitor compounds **A1** and **B4**, an in silico study was carried out to predict the binding poses in the binding site of BuChE (Figure 10), which showed good affinity. Regarding hybrid **A1** binding to the enzyme, the major interactions are through π–π stacking interactions, such as Trp82 from CAS with phenyl ring linked to the 4-position of the DHPM core, His438 from catalytic triad in the CAS with DHPM and 1,2,3-triazole cores, and Trp231, Phe329, and Tyr332 (from PAS) with a phenyl ring linked to 1,2,3-triazole moiety. Compound **B4** binds the amino acid residues of the enzyme via π–π stacking, such as Trp231 with a phenyl ring linked to the 1,2,3-triazole in the 5-position of the DHPM core and Trp82 from CAS with DHPM and its phenyl substituent of the 4-position. Asp70 and Tyr332 from PAS and Val288 from the acyl pocket bind to the isatin core. Ser72 and/or Gln71 could establish an H-bond with the carbonyl group of the isatin unit. Additionally, general Van der Waals interactions were also observed between the inhibitors and the enzyme. Hybrid **B4** exhibits mixed inhibition, binding at both the CAS and the PAS sites but with an apparent higher affinity for the PAS of BuChE rather than th CAS of BuChE.

**Figure 11:**
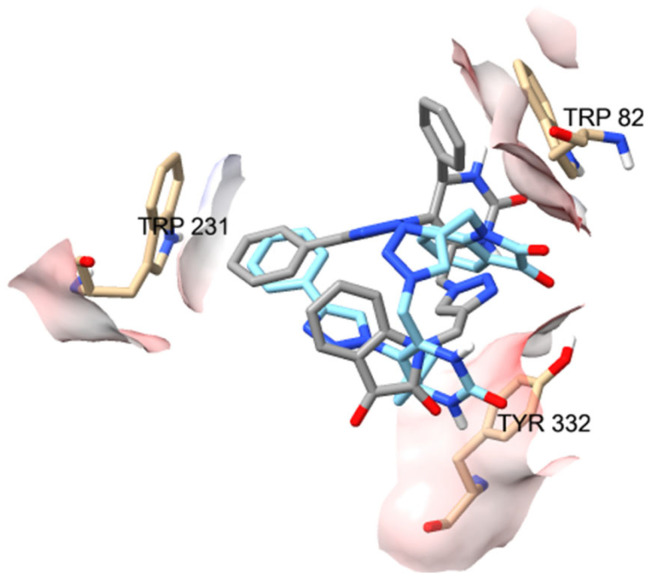
Interaction profiles of the two types of ten best-docked poses for **B4** against the BuChE enzyme (PDB ID 4AQD). The gray pose corresponds to the 1st, 2nd, 5th, 6th, and 10th best-docked poses, while the blue pose corresponds to the 3rd, 4th, 7th, 8th, and 9th.

However, even more relevant seems to be the π–π stacking interactions with the CAS residue (Trp82) and with the two PAS residues (Trp231 and Tyr332). It is observed that in the ten best poses of **B4** against the BuChE enzyme (PDB ID 4AQD), these poses oscillate between the pose represented in gray (five conformers) and in blue (five conformers) in Figure 11. As can be seen in Figure 11, for the Trp82 residue in the pose represented in gray, the mentioned interaction occurs with the phenyl ring attached to the DHPM core at position 4, while, in the blue pose, the interaction occurs with the isatin moiety. The opposite occurs with the Tyr332 residue in the two poses represented, gray and blue, in Figure 11.

### 2.3. STD-NMR Studies

In order to gain an insight into the interaction of our 1,2,3-triazole-DHPM hybrids with eqBuChE, we performed an STD-NMR study on the most potent compounds **A1** and **B4**. STD-NMR is a very useful validation technique for docking studies, which we have used previously with success [15,29,32].

#### 2.3.1. STD-NMR of Hybrid A1

**Figure 12:**
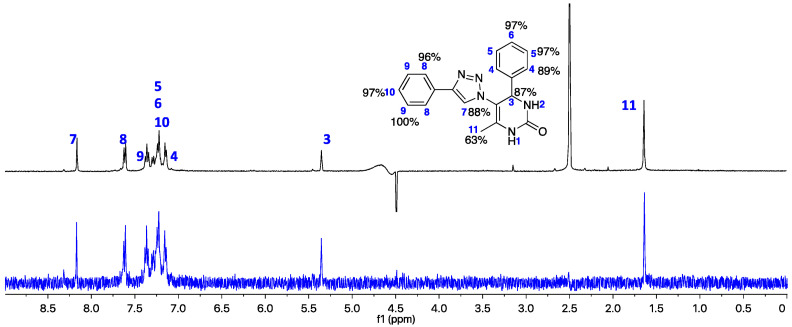
(Black line) Reference ^1^H NMR spectrum of hybrid **A1** (2.5 mM) with eqBuChE enzyme (2.5 μM). (Blue line): the corresponding STD-NMR spectrum with 3 s of saturation. The H9 proton was set to 100%. The NMR spectra were carried out in the mixture of solvents, DMSO-*d_6_* and D_2_O, at 25 °C.

The results obtained in the STD-NMR experiment of hybrid **A1** with the eqBuChE enzyme are shown in Figure 12 (the graph of the STD amplification factor as a function of saturation time (s) is shown in the Supplementary Materials, Figure S1); moreover, it can be seen that all the hydrogens interact with the amino acid residues in the enzyme’s binding pocket, with the exception of H1 and H2 of the N-H of the DHPM core. The aromatic hydrogens of the two phenyl rings show an attenuation of around 89–100%, showing a strong interaction with the enzyme. These results are completely in line with the prediction of the docking study, which shows the interaction of these aromatic rings with Trp82 (CAS), Tyr332 (PAS), Trp231, and Phe329. The H3 and H7 hydrogens of the main nuclei 1,2,3-triazole and DHPM showed very good attenuation values of 87 and 88%, respectively. This confirms the predicted interaction of His438 from the catalytic trio in the CAS with these cores.

#### 2.3.2. STD-NMR of Hybrid B4

**Figure 13:**
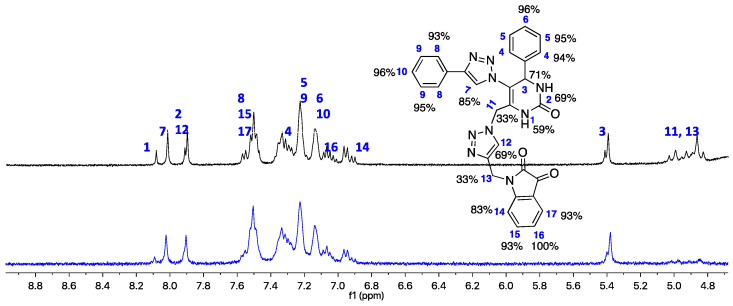
(Black line) reference ^1^H NMR spectrum of hybrid **B4** (2.5 mM) with eqBuChE enzyme (2.5 μM). (Blue line): the corresponding STD-NMR spectrum with 4 s of saturation. The H16 proton was set to 100%. The NMR spectra were carried out in the mixture of solvents, DMSO-*d_6_* and D_2_O, at 25 °C.

The results obtained for the STD-NMR experiment for the most potent inhibitor against eqBuChE are shown in Figure 13 (the graph of the STD amplification factor as a function of saturation time (s) is shown in the Supplementary Materials, Figure S2). All the hydrogens of the hybrid interact with the amino acid residues of the enzyme; however, the H11 and H13 of the CH_2_ groups showed a moderate attenuation value of 33%. Once again, and according to the epitope intensities shown in Figure 10, the aromatic hydrogens are the most affected by the amino acid residues, showing high attenuations of around 93–96%; as suggested by the docking study, these aromatic hydrogens interact with Trp231 and Trp82 in PAS and CAS, respectively. The H1–H3 hydrogens from the DHPM core showed 59–71% attenuation and may interact with Trp82, as predicted. The H15–H17 hydrogens show 93–100% attenuation, which indicates that they are very close to the enzyme; in fact, the docking study suggests that these hydrogens are close to the Asp70 and Tyr332 in PAS and Val288 in the acyl pocket.

### 2.4. In Vitro Antioxidant Assays

To assess the antioxidant activity of compounds **A1**–**3** and **B1**–**5**, the in vitro methods DPPH, ABTS, and FRAP were used.

All compounds evaluated at concentrations between 6 and 200 µM presented null values of antioxidant activity when using the DPPH and ABTS methods. It is thus concluded that, at least in this concentration range, these compounds do not have the capacity to scavenge free radicals (nor DPPH or ABTS radicals).

**Figure 14:**
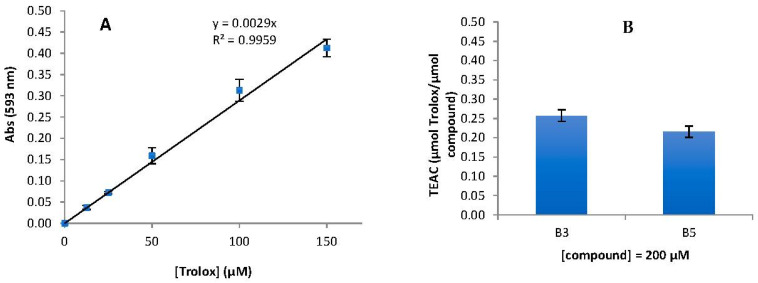
(**A**) Calibration curve of Trolox; (**B**) TEAC obtained from FRAP method for B3 and B5 compounds at 200 µM.

In the FRAP method, compounds **A1**–**3**, **B1, B2,** and **B4** also have null values of antioxidant capacity. On the other hand, the compounds **B3** and **B5** presented Trolox Equivalent Antioxidant Capacity (TEAC) in the FRAP method, although much lower than the positive control Trolox (a soluble analog of Vitamin E), and the results can be observed in Figure 14. At 200 μM, the highest concentration tested, TEACs of 0.257 and 0.216 were found for **B3** and **B5**, respectively, compared to Trolox, revealing that these compounds have some ability to reduce Fe(III) to Fe(II).

### 2.5. Toxicity Assay In Vivo

*Artemia salina* was used for in vivo general toxicity assessment of the compounds **A1**–**3** and **B1**–**5**. The results are shown in Table 3.

The test with K_2_Cr_2_O_7_ showed an LC_50_ of 23.6 mg/L. This validates the method as it is within the reference range (CI = 95% {20.7–38.3 mg/L}). Relative to the 1,2,3-triazole-dihydropyrimidinone hybrids tested, the compounds **A1**–**3**, **B1**, and **B3**–**5** showed no toxicity to *Artemia salina* in the concentration range tested. It should be noted that, for these compounds, no toxicity (NOEC) was observed at 200 μM concentration. The compound **B1** presented 37 ± 3% mortality at 200 μM concentration (data showed in Supplementary Materials, Figures S3–S11), which means that the LC_50_ will be >200 μM, also revealing a low toxicity.

### 2.6. SwissADME Calculations

Our study involved predicting the physicochemical properties of all examined hybrids using SwissADME [33]. Remarkably, all calculated results adhered to Lipinski’s rules [34], except for hybrids **B2**, **B4**, and **B5**, which slightly exceeded the specified MW limit. Encouragingly, none of these compounds were flagged as Pan-Assay Interference Compounds (PAINS), indicating no adverse interactions with multiple targets that could affect screening experiments negatively (Table 4). While all evaluated hybrids were predicted to be suitable for gastrointestinal absorption (GI), only hybrids **A1** and **A2** were anticipated to penetrate the blood–brain barrier (BBB). Most products exhibited moderate water solubility, with hybrid **A1** being notably soluble, while **B5** displayed poor solubility (Table 4). In fact, our best inhibitor **B4** does not permeate the BBB; however, this situation can be overcome by encapsulating it in nanoparticles of various types [35]. All hybrids, except **A3,** are estimated to be non-inhibitors of cytochrome P450 isoforms (CYP2D6 and CYP3A4), an important class of detoxification enzymes primarily found in the liver.

## 3. Materials and Methods

### 3.1. General Remarks

For carrying out this work, reagents were used as received. Iodide acetylthiocholine (ATCI), *S*-butyrylthiocholine iodide (BTCI), and 5,5′-dithiobis(2-nitrobenzoic acid) (DTNB) (Ellman’s reagent) were obtained from Sigma–Aldrich (St. Louis, MO, USA) and Alfa Aesar (Haverhill, MA, USA). The selected enzymes were purchased from Sigma–Aldrich: electric eel acetylcholinesterase (*ee*AChE) (E.C.3.1.1.7, Type VI-S, lyophilized powder, 500 U/2 mg), and equine serum butyrylcholinesterase (eqBuChE) (E.C. 3.1.1.8, lyophilized powder, 10.9 U/mg). The commercial inhibitors used were galantamine (TCI). 1,1-Diphenyl-2-picrylhydrazyl (DPPH), (2,2′-Azino-bis(3-ethylbenzothiazoline-6-sulfonic acid) diammonium salt) ABTS used to determine the radical-scavenging activity, was purchased from Fluka. TPTZ (2,4,6-Tris(2-pyridyl)-s-triazine) and *Trolox* (6-Hydroxy-2,5,7,8-tetramethylchroman-2-carboxylic acid) was purchased from BLDPharma. All other reagents and solvents used in the experiments were of analytical grade.

### 3.2. 1,2,3-Triazole-Dihydropyrimidinone Hybrids **A** and **B**

1,2,3-Triazole-Dihydropyrimidinone hybrids **A** and **B** were previously reported by Carreiro et al. [19]. ^1^H spectra data are included in the Supplementary Materials. The 8 hybrids evaluated were:

6-Methyl-4-phenyl-5-(4-phenyl-1,2,3-triazol-1-yl)-3,4-dihydropyrimidin-2-one (**A1**); 4-(4-Chlorophenyl)-6-methyl-5-(4-phenyl-1,2,3-triazol-1-yl)-3,4-dihydropyrimidin-2-one (**A2**); 4-(4-(Benzyloxy)phenyl)-6-methyl-5-(4-phenyl-1,2,3-triazol-1-yl)-3,4-dihydropyrimidin-2-one (**A3**); 4-Phenyl-5-(4-phenyl-1,2,3-triazol-1-yl)-6-((4-phenyl-1,2,3-triazol-1-yl)methyl)-3,4-dihydropyrimidin-2-one (**B1**); 6-((4-(4-Bromophenyl)-1,2,3-triazol-1-yl)methyl)-4-phenyl-5-(4-phenyl-1,2,3-triazol-1-yl)-3,4-dihydropyrimidin-2-one (**B2**); 6-((4-Cyclopropyl-1H-1,2,3-triazol-1-yl)methyl)-4-phenyl-5-(4-phenyl-1,2,3-triazol-1-yl)-3,4-dihydropyrimidin-2-one (**B3**); 1-((1-((2-Oxo-6-phenyl-5-(4-phenyl-1,2,3-triazol-1-yl)-1,2,3,6-tetrahydropyrimidin-4-yl)methyl)-1,2,3-triazol-4-yl)methyl)indoline-2,3-dione (**B4**); 4-(4-(Benzyloxy)phenyl)-5-(4-phenyl-1,2,3-triazol-1-yl)-6-((4-phenyl-1,2,3-triazol-1-yl)methyl)-3,4-dihydropyrimidin-2-one (**B5**).

### 3.3. Cholinesterase Inhibitory Assays

The enzymatic activity of *electrophorus electricus* AChE (*ee*AChE) and *equine serum* BuChE (eqBuCHE) was assessed using the Ellman colorimetric assay, with minor modifications. DMSO (final concentration of 1.25% in the cuvette) was used to prepare the stock solutions of the inhibitors (**A1**–**3** and **B1**–**5**). Enzyme kinetics were monitored using UV-Vis spectroscopy using 0.875 mM 5,5′-dithiobis(2-nitrobenzoic acid) (DTNB) (Ellman’s reagent) as a chromogenic agent. The reaction took place in a buffered medium (0.1 M phosphate buffer, pH 8.0), T = 25 °C, and was monitored for 125 s. To determine the percentage of inhibition ([I] = 100 µM), the substrate concentration (acetylthiocholine iodide for AChE and butyrylthiocholine iodide for BuChE) was set at 121 µM for AChE and 112 µM for BuChE. The results are shown in Table 1.

For compounds showing strong inhibition at 100 µM inhibition concentration, IC_50_ values were obtained. For this purpose, GraphPad Prism 8.02 software was used to plot %I vs. [I] (5 different concentrations) via a non-linear regression.

Methods of Dixon (Figure 5) and Cornish-Bowden (Figure 6) (1/V vs. [I] and [S]/V vs. [I]) were used for the visualization of the type of inhibition of the most active compound, hybrid **B4**. Calculation of the kinetic parameters (K_M_, V_max_) was accomplished using a nonlinear regression analysis (least squares fit) implemented in GraphPad Prism 8.01 software. The following equations were used for calculating the inhibition constants of the inhibitor obtained herein:

$$K_{m,app}=K_{M}\frac{1+\frac{\left[I\right]}{K_{ia}}}{1+\frac{\left[I\right]}{K_{ib}}}$$

$$V_{max,app}=\frac{V_{max}}{1+\frac{\left[I\right]}{K_{ia}}}$$

K_ia_: Inhibition constant for the interaction of the inhibitor with the free enzyme (E).

K_ib_: Inhibition constant for the interaction of the inhibitor with the complex enzyme-substrate (E-S).

### 3.4. In Silico Studies

Protein X-ray crystal structures were obtained from the Protein Data Bank (https://www.rcsb.org/, accessed on 7 October 2024), 6O4W and 4EY7 for AChE and 4AQD, 7Q1M and 4BDS for BuChE. All protein structures were determined at high resolution. The structures were inspected and assessed as adequate for docking, with resolutions of 2.35 Å, 2.35 Å, 2.5 Å, 2.79 Å, and 2.10 Å, respectively. The optimization of the 3D structures of the most promising inhibitors, **A1** and **B4**, as well as the positive controls (donepezil (PDB IDs 6O4W and 4EY7), β-alanine (PDB ID 4AQD), 3-(cyclohexylmethyl)amino-2-hydroxypropyl (PDB ID 7Q1M), tacrine (PDB ID 4BDS), and galantamine (PDB IDs 6O4W, 4EY7, 4AQD, 7Q1M and 4BDS) was carried out using the RDKit function MMFFOptimizeMolecule with the arguments mmffVariant = ‘MMFF94’ and maxIters = 5000 in Python (cite: Landrum, G. RDKit: Open-Source Cheminformatics Software. http://www.rdkit.org (2016)). Hydrogen atoms were added with Maestro software [Schrödinger, 2020]. Docking was then performed using extra precision Glide XP [Schrödinger, 2020] with extended sampling and the OPLS3e forcefield [36]. The software program OpenBabel (version 2.3.1) [37] was used to convert the mol2 files to PDBQT files. PDBQT files were used for docking to AChE (PDB IDs 604W and 4EY7) and BuChE (PDB IDs 4AQD, 7Q1M, and 4BDS) enzymes with AutoDock Vina (version 1.2.3) [38,39]. Water molecules, ions, and ligands were removed from all enzymes (604W, EY7, 4AQD, 7Q1M, 4BDS) prior to docking using the AutoDockTools (http://mgltools.scripps.edu/, accessed on 29 August 2024). The search space coordinates were AChE enzyme; 6O4W—Centre X: 89.341 Y: 84.453 Z: −5.628, and 4EY7—Centre X: −15.834 Y: −43.535 Z: 25.391; and BuChE enzyme; 4AQD—Centre X: 7.0 Y: −10.639 Z: −12.236, 7Q1M—Centre X: 19.553 Y: 42.576 Z: 41.06, and 4BDS—Centre X: 131.866 Y: 112.975 Z: −44.529, Dimensions X: 20.000 Y: 20.000 Z: 20.000. Ligand tethering of the AChE and BuChE enzymes was performed by regulating the genetic algorithm (GA) parameters, using 10 runs of the GA criteria. The docking binding poses were visualized with PyMOL Molecular Graphics System, Version 2.0 Schrödinger, LLC (New York, NY, USA), UCSF Chimera [40], and the Protein–Ligand Interaction Profiler (PLIP) web tool (https://plip-tool.biotec.tu-dresden.de/plip-web/plip, accessed on 7 October 2024) [41].

### 3.5. STD-NMR Experiments

The NMR spectroscopy experiments were performed on a Bruker Avance III 400 MHz spectrometer equipped with a 5 mm broadband (PABBO BB/19F-1H/D Z-GRD) resonance probe head. The STD-NMR experiments were realized according to our established method [29,32]. Basically, NMR and STD-NMR experiments were carried out with solvent suppression and a 10 ms spinlock filter after the 90◦ pulse to reduce residual signals from the protein. For selective saturation, cascades of Gaussian pulses with a length of 50 ms and 40–60 dB of attenuation were employed, with an interpulse delay of 1 ms [29,32]. The on-resonance and off-resonance frequencies were set to 0 and 12,000 Hz, respectively. STD-NMR controls were performed using the ligand itself. Blank experiments were performed to guarantee the absence of direct saturation of the ligand proton signals. The relaxation delay was properly adjusted so that the experiment time length was kept constant at 6.5 s. Water suppression at 1880 Hz (4.7 ppm) was conducted. Specifically, the saturation time to obtain the STD buildup curves was recorded at 0.25, 0.5, 1, 2, 3, 4, and 5 s [29,32]. A 5 μM eqBuChE solution was prepared in a D_2_O. Five mM stock solutions were prepared for hybrids **A1** and **B4**. Samples for NMR analysis were prepared by adding 300 μL of the ligand to a 300 μL enzyme solution. The final concentrations of ligand and enzyme were 2.5 µM and 2.5 mM, respectively.

### 3.6. Antioxidant Assays

#### 3.6.1. DPPH Antioxidant Assay

In the DPPH method, the antioxidants react with DPPH^•^ (2,2-diphenyl-1-picrylhydrazyl) (which has a strong violet color) and convert it into 2,2-diphenyl-1-picrylhydrazine (yellow in color). The colorimetric DPPH assay was carried out as described in [29]. The compounds **A1**–**3** and **B1**–**5** were evaluated through the DPPH assay against positive control, ascorbic acid.

In brief, 30 μL of the tested compounds (6–200 µM in MeOH + DMSO 0.5%) was mixed with 200 μL of DPPH (0.03 g/L in MeOH) in a 96-well plate in triplicate. After 30 min incubation at room temperature in the absence of light, the absorbance was measured at 517 nm using Microplate Spectrophotometry (TriStar^®^ S LB 942 model instrument, Berthold Technologies GmbH & Co.KG, Bad Wildbad, Germany). The free radical scavenging activity (DPPH^•^) results are expressed as a percentage of DPPH inhibition according to the following formula:

$$Inhibition\;(\%)=\frac{A_{blank}-A_{sample}}{A_{blank}}\times 100$$

where A_blank_ consists of MeOH + DMSO 0.5%) (30 μL) mixed with DPPH (200 μL) absorbance, and A_sample_ is the absorbance value for the added sample concentration compound mixed with DPPH.

#### 3.6.2. ABTS Antioxidant Assay

The ABTS (2,2′-Azino-bis(3-ethylbenzothiazoline-6-sulfonic acid) diammonium salt) test measures the relative capacity of antioxidants (hydrophilic and lipophilic) to eliminate ABTS generated in the aqueous phase (light green). ABTS^•^ is generated by the reaction of a strong oxidizing agent (e.g., potassium persulfate, K_2_S_2_O_8_) with the ABTS salt (dark green color). The assay was carried out using the modified method described in [42]. The compounds **A1**–**3** and **B1**–**5** were selected through the ABTS assay against the positive control, ascorbic acid.

In brief, 30 μL of the tested compounds (6–200 µM in MeOH + DMSO 0.5%) was mixed with 200 μL of ABTS (prepared by mixing 0.373mmol of ABTS and 0.125 mmol of K_2_S_2_O_8_ in MeOH over 24H stirring) in a 96-well plate in triplicate.

After 30 min incubation at room temperature in the absence of light, the absorbances were measured at 517 nm using Microplate Spectrophotometry (TriStar^®^ S LB 942 model instrument, Berthold Technologies GmbH & Co.KG, Bad Wildbad, Germany). The free radical scavenging activity (ABTS^•^) results are expressed as a percentage of ABTS inhibition according to the following formula:

$$Inhibition\;(\%)=\frac{A_{blank}-A_{sample}}{A_{blank}}\times 100$$

where A_blank_ consists of MeOH + DMSO 0.5% (30 μL) mixed with ABTS (200 μL) absorbance, and A_sample_ is the absorbance value for the added sample concentration compound mixed with ABTS.

#### 3.6.3. FRAP Antioxidant Assay

The Ferric Reducing Antioxidant Power (FRAP assay) evaluates antioxidant capacity by quantifying the reduction in the Fe(III)-2,4,6-Tris(2-pyridyl)-s-triazine (TPTZ) complex (light blue in color) to Fe(II)-TPTZ (dark blue in color), in an acidic medium (HCl), compared to a *Trolox* (6-hydroxy-2,5,7,8-tetramethylchroman-2-carboxylic acid) standard (analog of vitamin E).

The assay was carried out as described in [43]. The compounds **A1**–**3** and **B1**–**5** were selected through the FRAP assay against positive control and water-soluble analogs of vitamin E and *Trolox*.

In brief, 30 μL of the tested compounds (6–200 µM in MeOH + DMSO 0.5%) was mixed with 200 μL of the FRAP reagent (obtained from a mixture of 0.3 M acetate buffer, 10 mM TPTZ solution, and 20 mM ferric chloride solution in a ratio of 10:1:1 (*V*/*V*), respectively) in a 96-well plate in triplicate. After 10 min incubation at room temperature in the absence of light, the absorbances were measured at 593 nm using Microplate Spectrophotometry (TriStar^®^ S LB 942 model instrument, Berthold Technologies GmbH & Co.KG, Bad Wildbad, Germany). The results of the Fe(II)-TPTZ complex formation are expressed as *Trolox* Equivalent Antioxidant Capacity (TEAC) according to the following formula:

$$TEAC=\frac{c(TE)}{c(compound)}$$

where *c*(TE) is the concentration of *Trolox* and *c*(compound) is the concentration of compound tested, the *c*(TE) was determined according to the following formula:

$$c(TE)=\frac{A_{1}-A_{0}}{Slope}$$

where A_0_ consists of the solvent (MeOH + DMSO 0.5%) (30 μL) mixed with FRAP solution (200 μL) absorbance, A_1_ is the absorbance value for the added sample concentration compound mixed with FRAP solution, and the slope was obtained from the calibration curve of *Trolox* (positive control) (slope = 0.0029 with *R*^2^ = 0.99), Figure 14.

### 3.7. Artemia Salina Lethal Toxicity Assay

The ARTOXKIT M protocol was used. The percentage of dyed nauplii of *Artemia salina*, grown in the presence of variable concentrations of the inhibitor compounds (**A1**–**3** and **B1**–**5**) (0.01 to 100 µM).

Saline water with 1% DMSO was used as a blank. After 24 h incubation at 25 °C, the LD_50_ values were determined.

In parallel, a K_2_Cr_2_O_7_ test was performed as quality control.

### 3.8. Statistical Analysis

All experimental results are shown as the mean ± SD. Origin 9.0 software (OriginLab, Northampton, MA, USA), GraphPrism^®^ software (version 9.2, 64-bit) and Microsoft^®^ Excel^®^ (for Microsoft 365 MSO, version 2307 Build 16.0.16626.20198, 64-bit) were used for drawing and data analysis in this paper.

## 4. Conclusions

In conclusion, hybrids **A** and **B** were evaluated as inhibitors of *ee*AChE and eqBuChE and did not inhibit *ee*AChE (IC_50_ values of >100.0 µM). Five (**A1**, **A2**, **B1**, **B3**, and **B4**) of the eight hybrids evaluated proved to be eqBuChE inhibitors displaying IC_50_ values in the range of 1.0–58.0 µM. Hybrid **B4** was the most potent inhibitor, probably due to the presence of two 1,2,3-triazole rings linked to the core DHPM unit, in addition to an isatin ring as a substituent (both 1,2,3-triazole and isatin are potent pharmacophores). However, hybrid analogs **B1** and **B3** showed weaker inhibition than hybrids **A1** and **A2** (having only a single 1,2,3-triazole ring linked to the DHPM). Additionally, the latter compounds were predicted using SwissADME to be capable of permeating the BBB. Hybrid **A1** showed an IC_50_ of 12 ± 2 µM, which is very similar to galantamine. Hybrid **B4** showed the most potent inhibitory activity, with an IC_50_ value = 1.0 ± 0.1 μM, more active than the approved drug galantamine (IC_50_ = 10 μM). It can be concluded that the isatin substituent is the key to obtaining good anticholinesterase activity. Using the Cornish-Bowden method, it was concluded that hybrid **B4** shows mixed inhibition.

Both in silico and STD NMR studies confirm the strong binding of the inhibitors to the enzyme (BuChE) through π–π stacking and other interactions (such as Van der Waals) between the amino acid residues and the aromatic and heterocyclic rings of the inhibitors. Additionally, **B4** presented no toxicity in *Artemia salina* in the concentration range tested. This makes the **B4** hybrid the one with the best potential for future pharmacological application in Alzheimer’s disease.

The compounds **A1**–**3** and **B1**–**5** did not show significant antioxidant activity for DPPH and ABTS, with no capacity for radical scavenging. The compounds **B3** and **B5** showed some potential to reduce Fe(III) to Fe(II) using the FRAP method. Despite their low antioxidant and inhibitory activities, these compounds may hold potential as pharmacological agents for other therapeutic applications due to their low toxicity.

## Figures and Tables

**Table 2:** Calculated binding affinities (Score) upon docking the selected compounds, **A1** and **B4**, and positive controls against AChE and BuChE.

		Compounds		Positive Controls				
		A1	B4	Donepezil ^2^	β-alanine ^3^	HPD ^3^	Tacrine ^3^	Galantamine ^2,3^
Software ^1^	ChE (PDB ID)	Score (kcal/mol) ^4^						
Glide Xp	AChE (6O4W)	−3.72	−8.99	−15.08	−5.29	-	-	-
	BuChE (4AQD)	−7.80	−9.95	−7.04	−5.00	-	-	-
AutoDock Vina	AChE (6O4W)	−11.75	−11.13	−11.07	-	-	-	−9.89
	AChE (4EY7)	−11.63	−11.21	−11.63	-	-	-	−9.76
	BuChE (4AQD)	−10.58	−12.57	-	−3.967	-	-	−8.60
	BuChE (7Q1M)	−10.62	−13.28	-	-	−9.81	-	−8.72
	BuChE (4BDS)	−10.96	−12.57	-	-	-	−8.28	−8.98

^1^ Used for molecular docking. ^2^ Against AChE. ^3^ Against BuChE. ^4^ Calculated binding affinity.

**Table 3:** *Artemia salina* results for the compounds **A1**–**3** and **B1**–**5** expressed as LC_50_, NOEC (No Observed Effect Concentration), and LOEC (Lowest Observed Effect Concentration) parameters. Reference test: K_2_Cr_2_O_7_ (mg/L).

	K_2_Cr_2_O_7_	A1	A2	A3	B1	B2	B3	B4	B5
LC_50_	23.6 mg/L	-	-	-	-	>200 μM	-	-	-
NOEC	10 mg/L	200 μM	200 μM	200 μM	200 μM	1 μM	200 μM	200 μM	200 μM
LOEC	18 mg/L	-	-	-	-	100 μM	-	-	-

**Table 4:** Calculated ADME properties for all hybrids.

Hybrid	MW ^1^ (g/mol)	MLOGP	LogS	HBA ^2^	HBD ^3^	TPSA ^4^	GI	BBB	PAINS # Alerts	CYP2D6/CYP3A4 ^5^
**A1**	331.37	2.36	−3.62	3	2	71.84	High	Yes	0	No ^6^
**A2**	365.82	2.86	−4.21	3	2	71.84	high	yes	0	No ^6^
**A3**	437.49	3.13	−5.01	4	2	81.07	high	no	0	Yes ^7^
**B1**	474.52	2.95	−4.79	5	2	102.55	high	no	0	No ^6^
**B2**	553.41	3.51	−5.70	5	2	102.55	high	no	0	No ^6^
**B3**	438.48	2.59	−3.80	5	2	102.55	high	no	0	No ^6^
**B4**	557.56	1.79	−4.43	7	2	139.93	high	no	0	No ^6^
**B5**	580.64	3.59	−6.17	6	2	111.78	high	no	0	No ^6^

^1^ Molecular weight. ^2^ Number of hydrogen bond acceptors. ^3^ Number of hydrogen bond donors. ^4^ Topological surface area (Å^2^). ^5^ Cytochrome P450 isoform inhibitors. ^6^ Non-inhibitor. ^7^ Inhibitor.

## Data Availability

The original contributions presented in the study are included in the article/Supplementary Materials, and further inquiries can be directed to the corresponding author/s.

## Supplementary Materials

The following supporting information can be downloaded at: https://www.mdpi.com/article/10.3390/ijms252011153/s1.
