# Supplementary material for: Assessing the Potential of 1,2,3-Triazole-Dihydropyrimidinone Hybrids Against Cholinesterases: In Silico, In Vitro, and In Vivo Studies
Source: Int J Mol Sci. 2024 Oct 17;25(20):11153. doi: 10.3390/ijms252011153 (PMC11508620; doi:10.3390/ijms252011153)
Supplement: Supplementary file 1 [file ijms-25-11153-s001.zip › ijms-3242354-supplementary.pdf]

Supporting Information

Assessing the Potential of 1,2,3-Triazole-Dihydropyrimidinone Hybrids against Cholinesterases: In Silico, In Vitro and In Vivo studies

Carlos M. Gastalho [1,2,3], Ana M. Sena [4], Óscar López [5], José G. Fernández-Bolaños [5], Alfonso T. García-Sosa [6], Florbela Pereira[7], Célia M. Antunes [2,3,8], Ana R. Costa [2,3,8], Anthony J. Burke [1,9,10,11] and Elisabete P. Carreiro [1,*]

1 LAQV-REQUIMTE, Institute for Research and Advanced Training (IIFA), University of Évora, Rua Romão Ramalho, 59, 7000-671 Évora, Portugal
2 Institute of Earth Sciences, Institute of Research and Advanced Training, University of Évora, 7000-671 Évora, Portugal
3 Academic Clinical Center of Alentejo, C-TRAIL, Rua Romão Ramalho, 59, 7000-671 Évora, Portugal
4 Department of Chemistry and Biochemistry, School of Sciences and Technologies, University of Évora, Rua Romão Ramalho 59, 7000-671 Évora, Portugal
5 Departamento de Química Orgánica, Facultad de Química, Universidad de Sevilla, Apartado 1203, E-41071 Seville, Spain
6 Institute of Chemistry, University of Tartu, Ravila 14 A, Tartu 50411, Estonia. ORCiD 0000-0003-0542-4446
7 LAQV REQUIMTE, Department of Chemistry, NOVA School of Science and Technology, Universidade Nova de Lisboa, 2829516 Caparica, Portugal
8 Department of Medical and Health Sciences, School of Health and Human Development, University of Évora, Rua Romão Ramalho 59, 7000-671 Évora, Portugal
9 Faculty Pharmacy, University of Coimbra, Pólo das Ciências da Saúde, Azinhaga de Santa Comba, 3000-548 Coimbra, Portugal
10 Departamento de Química, Coimbra Chemistry Centre-Institute of Molecular Sciences (CQC-IMS), University of Coimbra, 3004-535 Coimbra, Portugal.
11 Center for Neurosciences and Cellular Biology (CNC), Polo I, Universidade de Coimbra Rua Larga Faculdade de Medicina, Polo I, 1ºandar 3004–504, Coimbra Portugal.
* Correspondence: betepc@uevora.pt (E.P.C.)

Contents: **pages**

1. ^{1}H NMR spectra of 1,2,3-triazole-dihydropyrimidinone hybrids: General Remarks 1
1.1. Hybrids A1-3 2-3
1.2. Hybrids B1-5 3 - 4
2. STD-NMR Experiment 6
3. Toxicity assay *in vivo* 7

1. ^{1}H NMR spectra of 1,2,3-triazole-dihydropyrimidinone hybrids: General Remarks

The eight 1,2,3-triazole-dihydropyrimidinone hybrids, **A1-3** and **B1-5** the target molecules of this work, were characterized by ^{1}H NMR to verify their structures. ^{1}H NMR spectra were recorded with a Bruker Avance III at 400 MHz. Chemical shifts were quoted in parts per million (ppm) and referenced to the appropriate solvent peak.

1.1. Hybrids A1-3:

1.1.1. 6-Methyl-4-phenyl-5-(4-phenyl-1,2,3-triazol-1-yl)-3,4-dihydropyrimidin-2-one A1;

^{1}H NMR (400 MHz, DMSO-d_6):

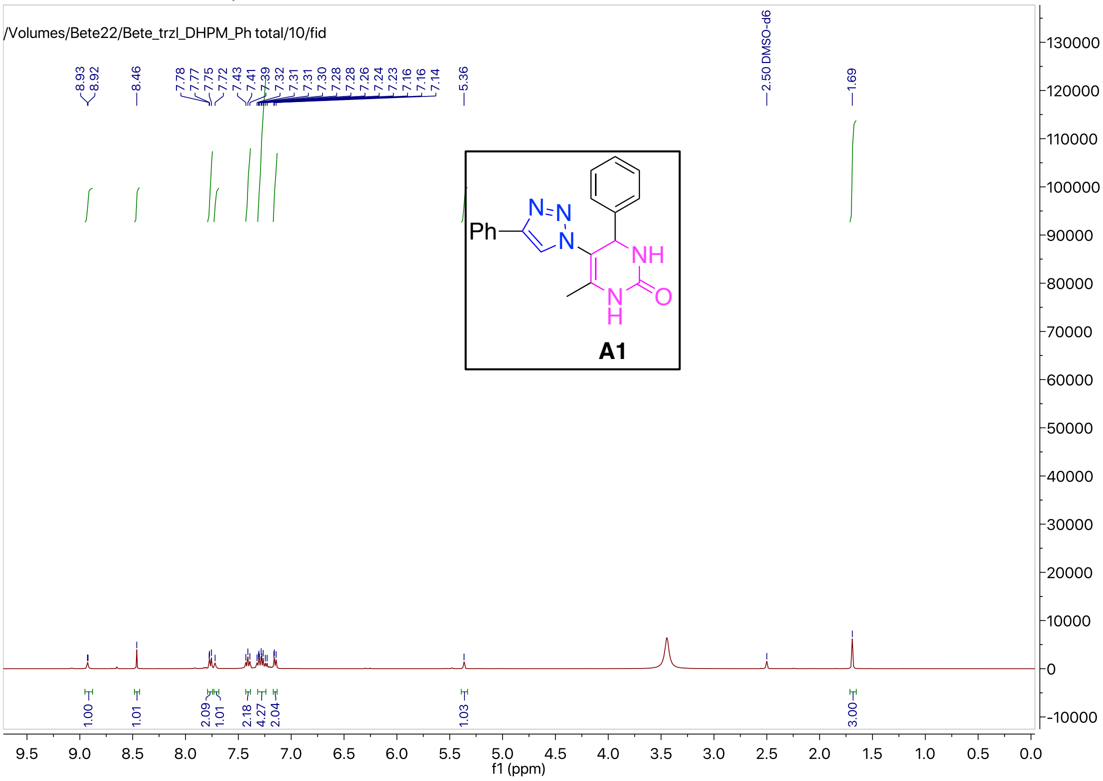

1.1.2. 4-(4-Chlorophenyl)-6-methyl-5-(4-phenyl-1,2,3-triazol-1-yl)-3,4-dihydropyrimidin-2-one (A2); ^{1}H NMR (400 MHz, DMSO-d_6):

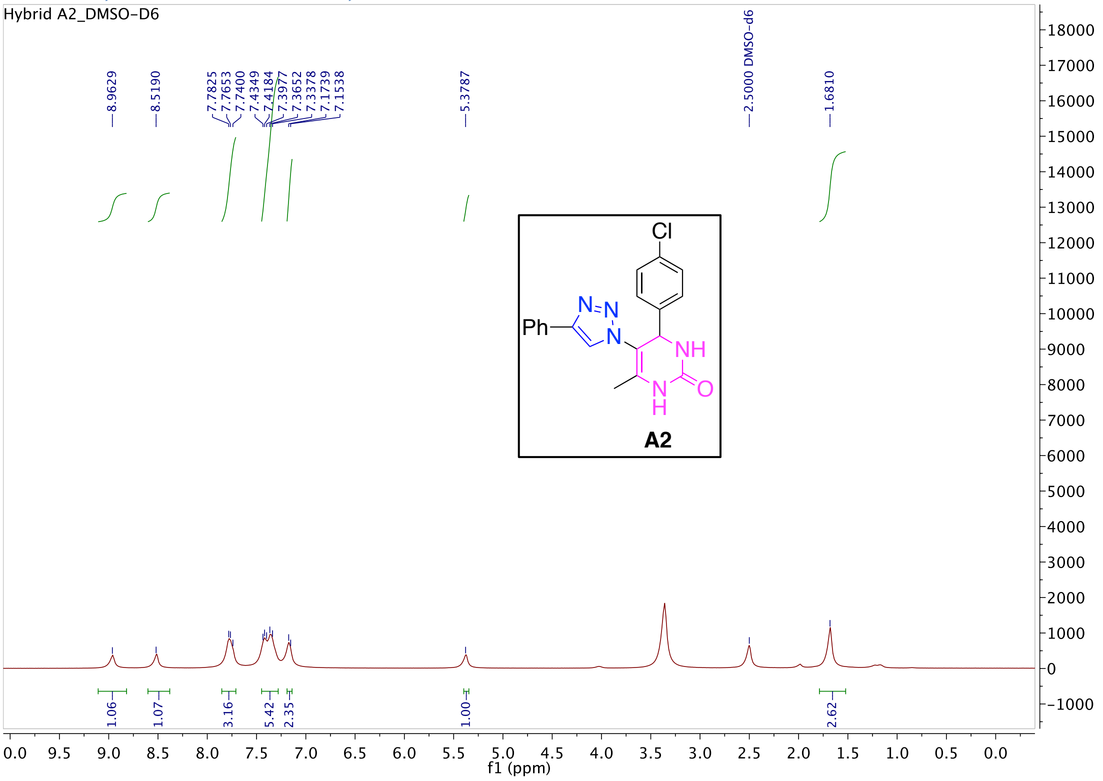

1.1.3. 4-(4-(Benzyloxy)phenyl)-6-methyl-5-(4-phenyl-1,2,3-triazol-1-yl)-3,4-dihydropyrimidin-2-one (A3); ^{1}H NMR (400 MHz, DMSO-d_6):

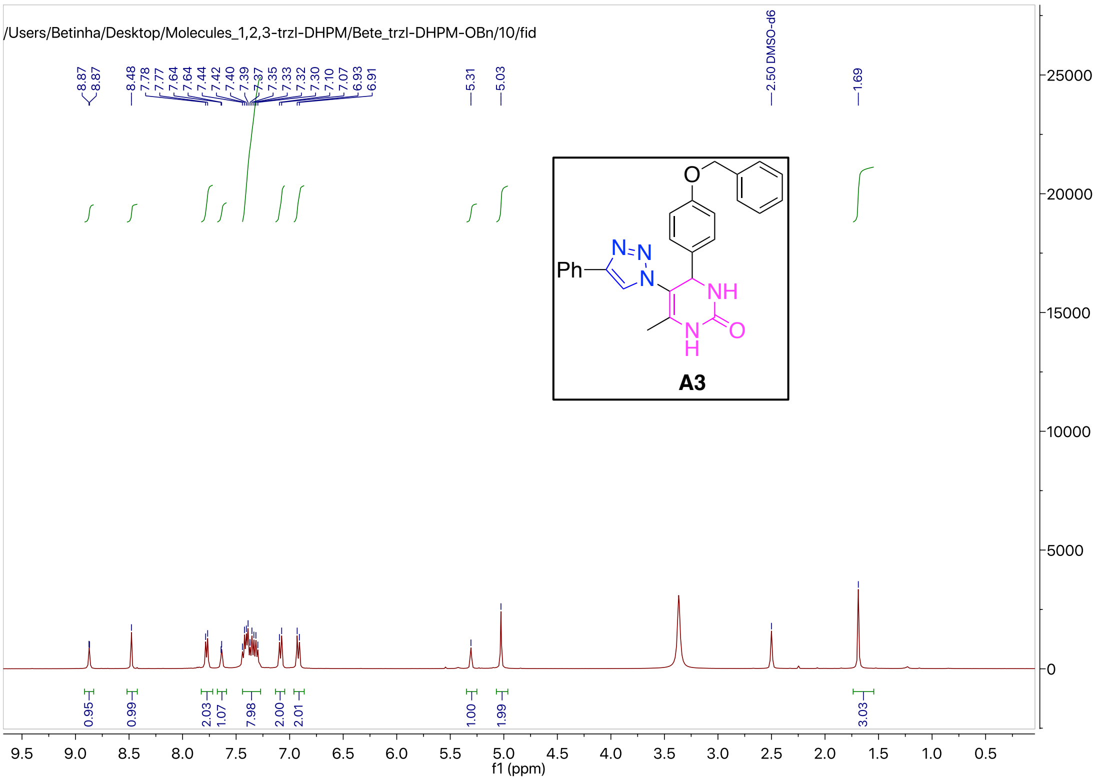

1.2. Hybrids B1-5:

1.2.1. 4-Phenyl-5-(4-phenyl-1,2,3-triazol-1-yl)-6-((4-phenyl-1,2,3-triazol-1-yl)methyl)-3,4-dihydropyrimidin-2-one (B1); ^{1}H NMR (400 MHz, DMSO-d_6):

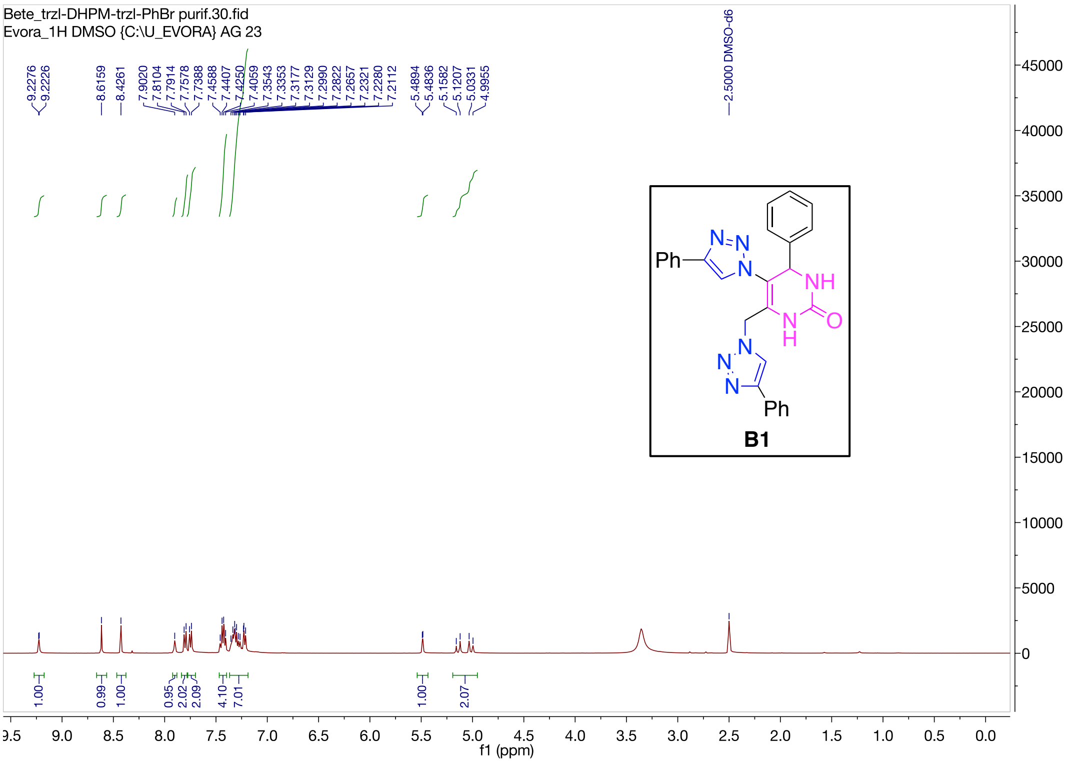

1.2.2. 6-((4-(4-Bromophenyl)-1,2,3-triazol-1-yl)methyl)-4-phenyl-5-(4-phenyl-1,2,3-triazol-1-yl)-3,4-dihydropyrimidin-2-one (B2); ^{1}H NMR (400 MHz, DMSO-d_6):

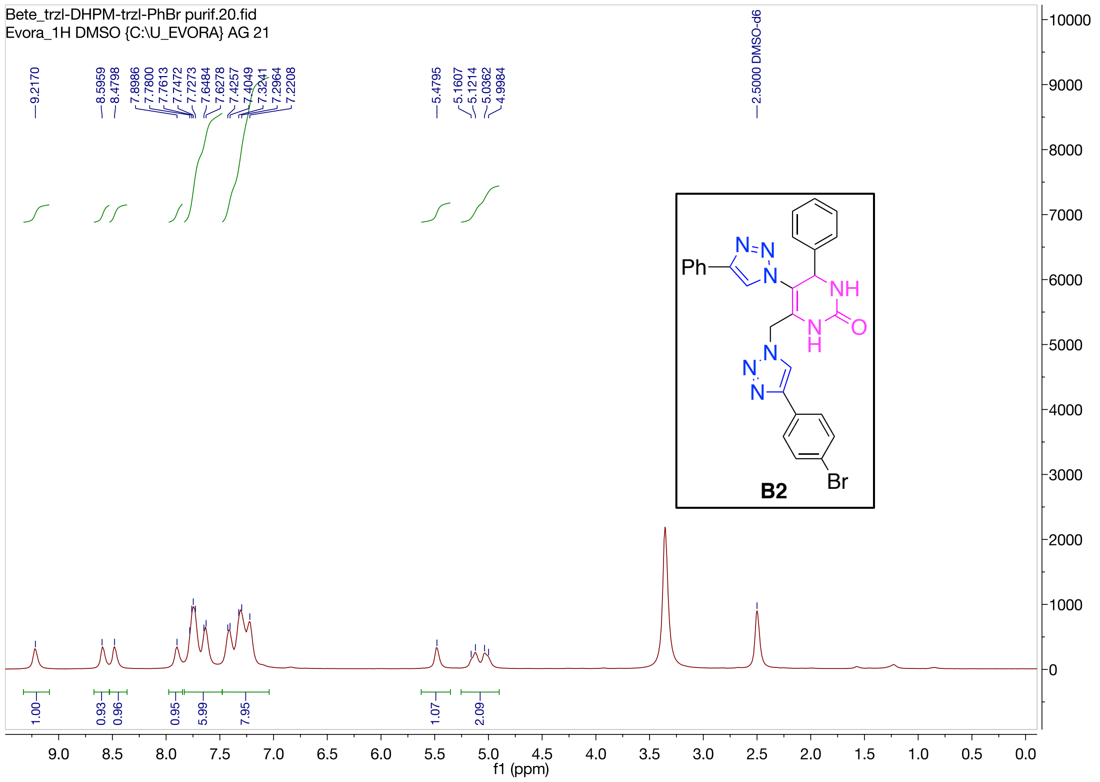

1.2.3. 6-((4-Cyclopropyl-1H-1,2,3-triazol-1-yl)methyl)-4-phenyl-5-(4-phenyl-1,2,3-triazol-1-yl)-3,4-dihydropyrimidin-2-one (B3); ^{1}H NMR (400 MHz, DMSO-d_6):

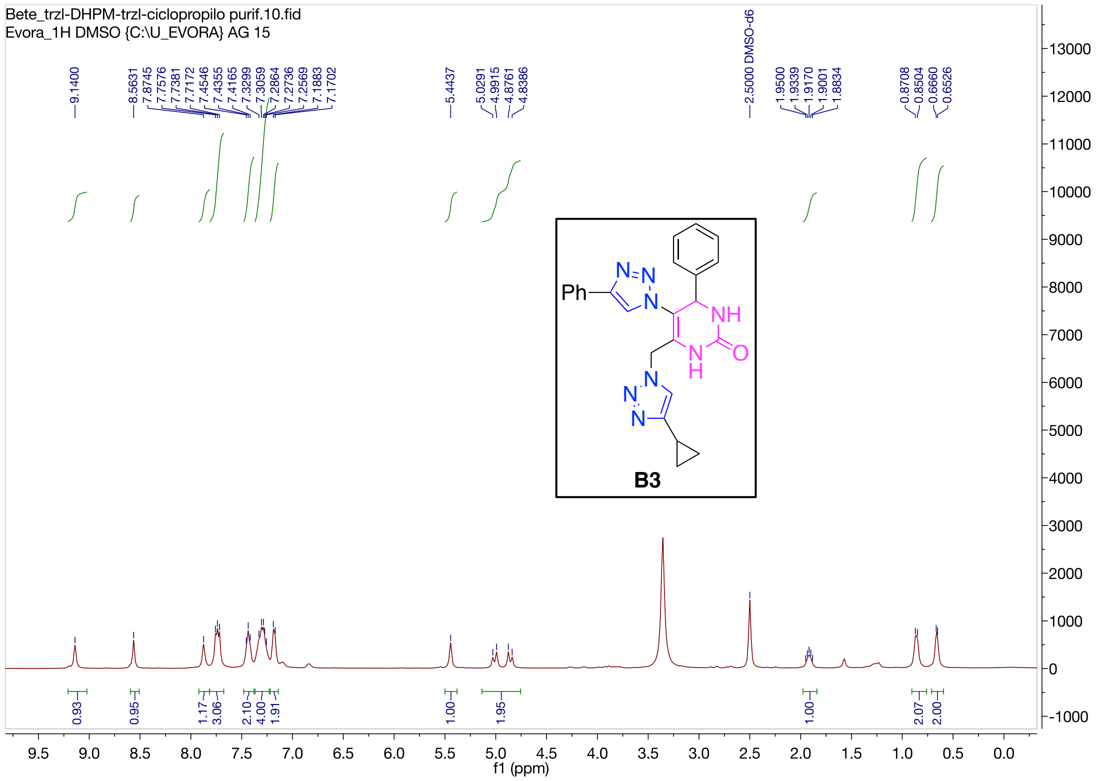

1.2.4. 1-((1-((2-Oxo-6-phenyl-5-(4-phenyl-1,2,3-triazol-1-yl)-1,2,3,6-tetrahydropyrimidin-4-yl)methyl)-1,2,3-triazol-4-yl)methyl)indoline-2,3-dione (B4); ^{1}H NMR (400 MHz, DMSO-d_6):

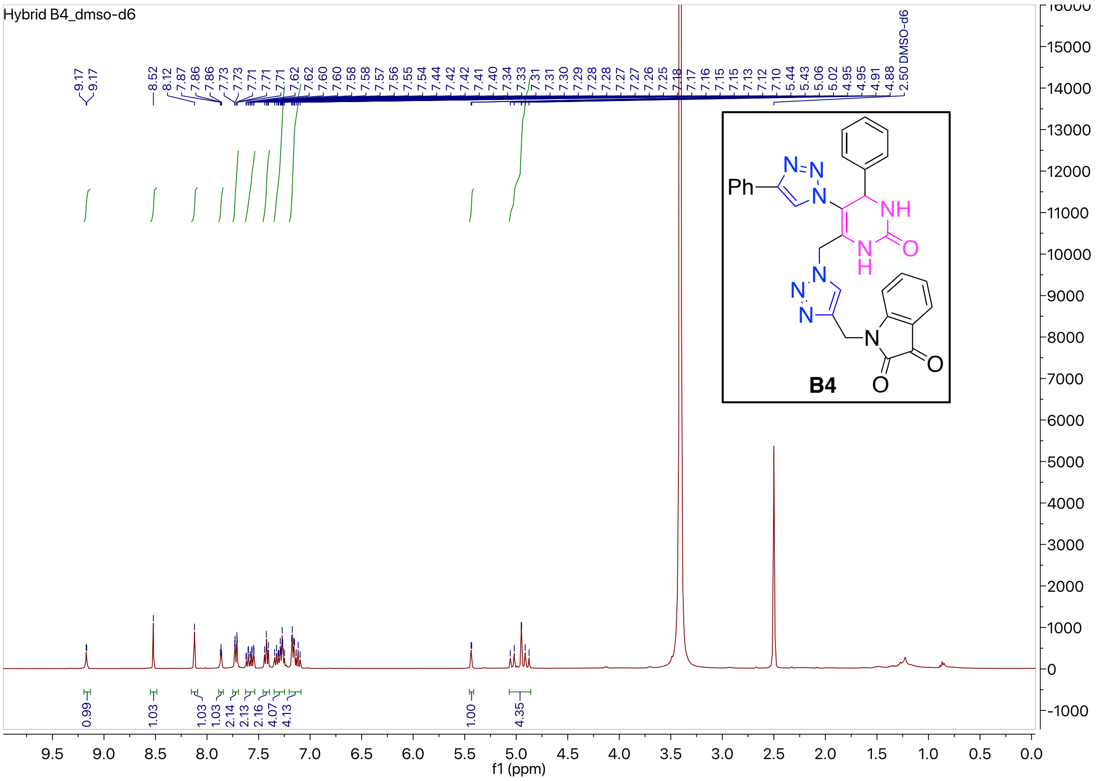

1.2.5. 4-(4-(Benzyloxy)phenyl)-5-(4-phenyl-1,2,3-triazol-1-yl)-6-((4-phenyl-1,2,3-triazol-1-yl)methyl)-3,4-dihydropyrimidin-2-one (B5); ^{1}H NMR (400 MHz, DMSO-d_6):

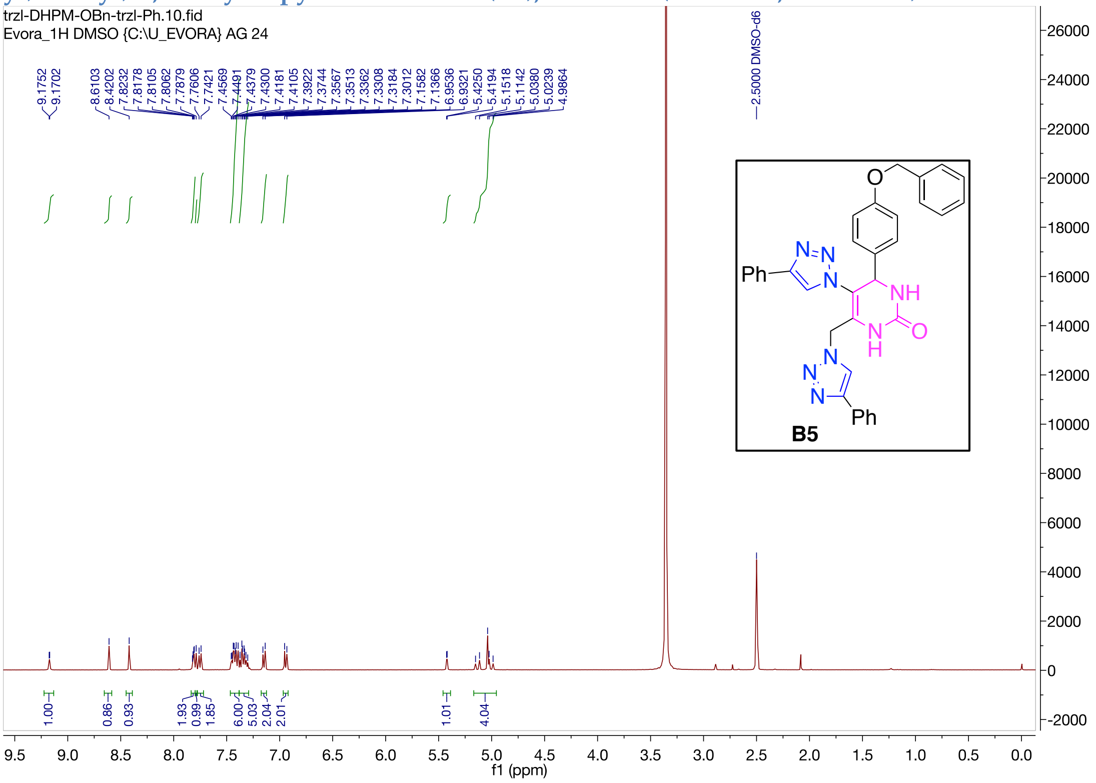

2. STD-NMR Experiment

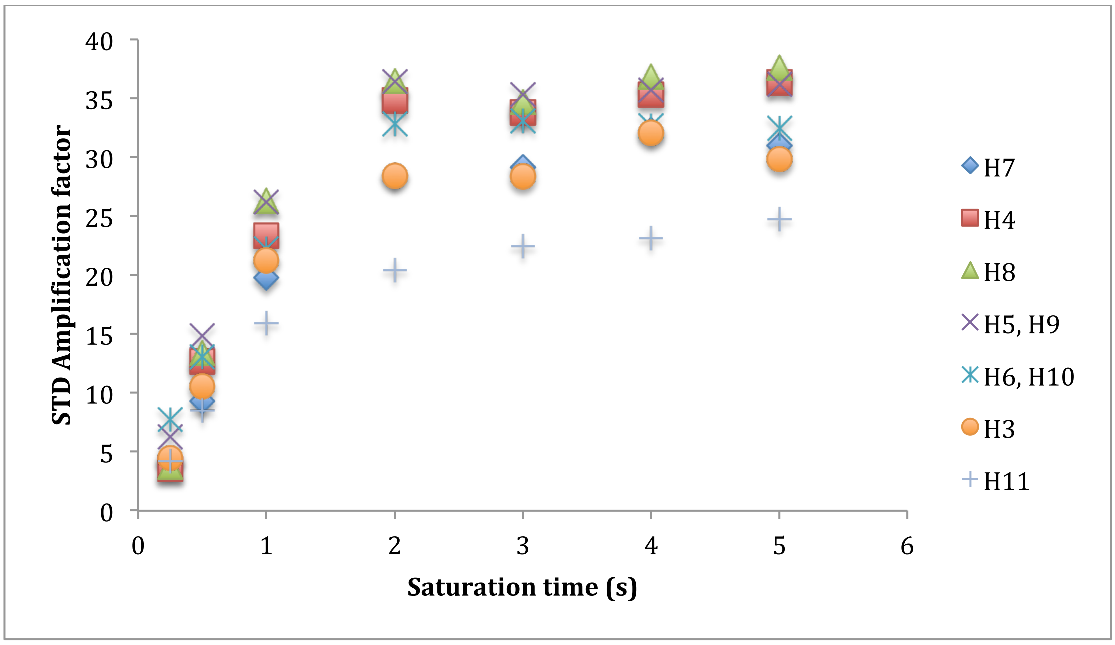

Figure S1. STD amplification factor as a function of saturation time (s) for hybrid **A1**.

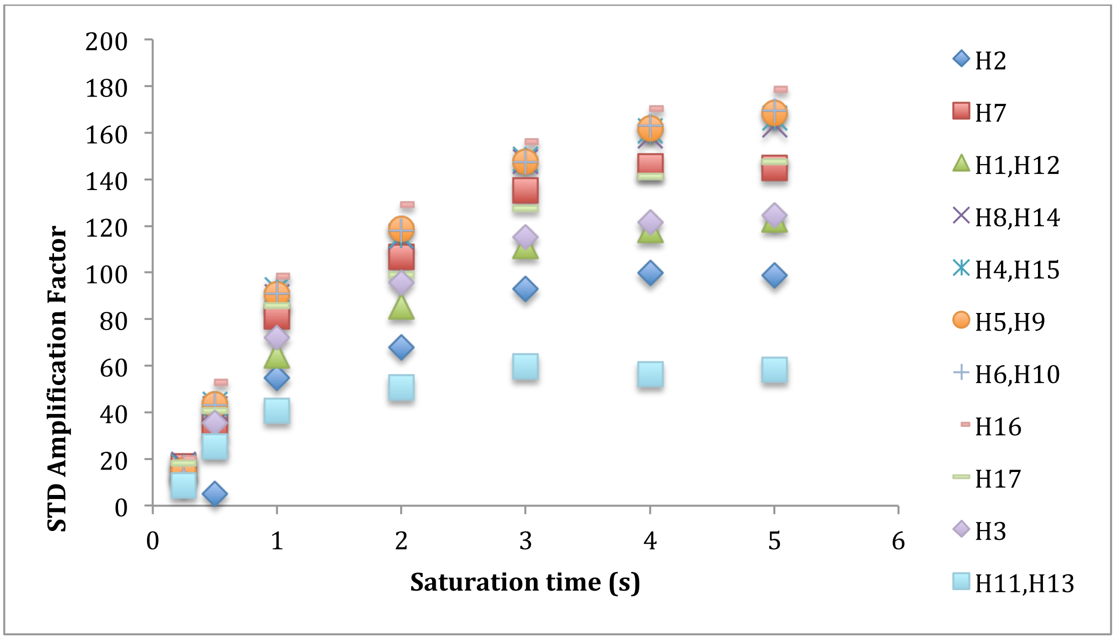

Figure S2. STD amplification factor as a function of saturation time (s) for hybrid **B4**.

3. Toxicity assay in vivo

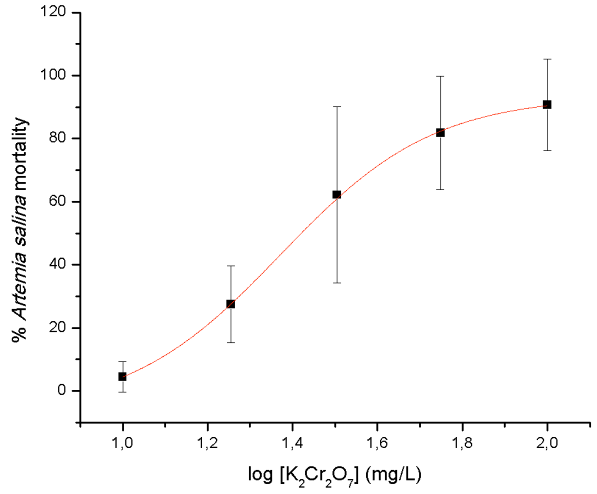

Figure S3. Mortality (%) of artemia salina *vs* log[$K_2Cr_2O_7$] – dose–response curves.

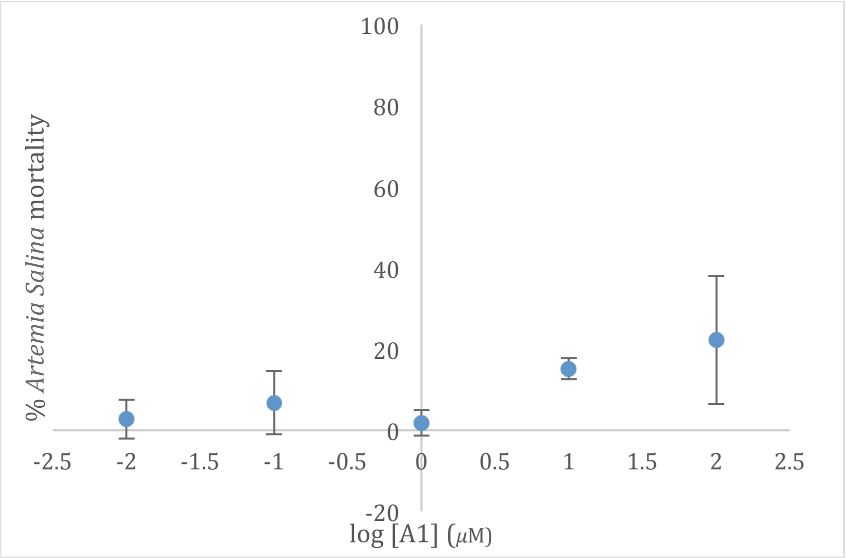

Figure S4. Mortality (%) of artemia salina *vs* log[**A1**] – dose–response curves.

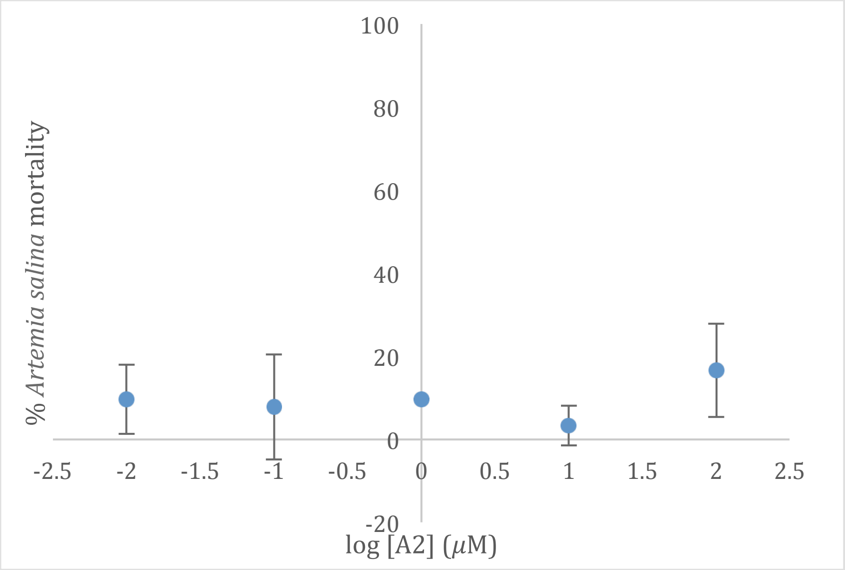

Figure S5. Mortality (%) of artemia salina *vs* log[**A2**] – dose–response curves.

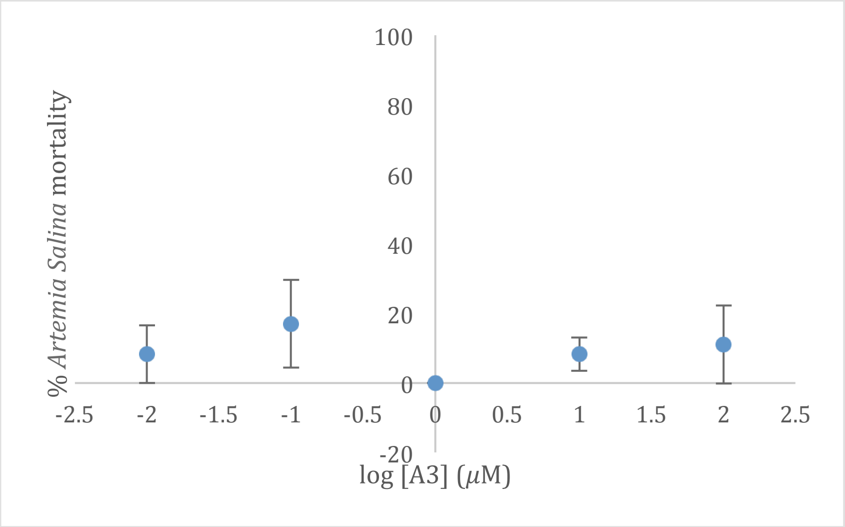

Figure S6. Mortality (%) of artemia salina *vs* log[**A3**] – dose–response curves.

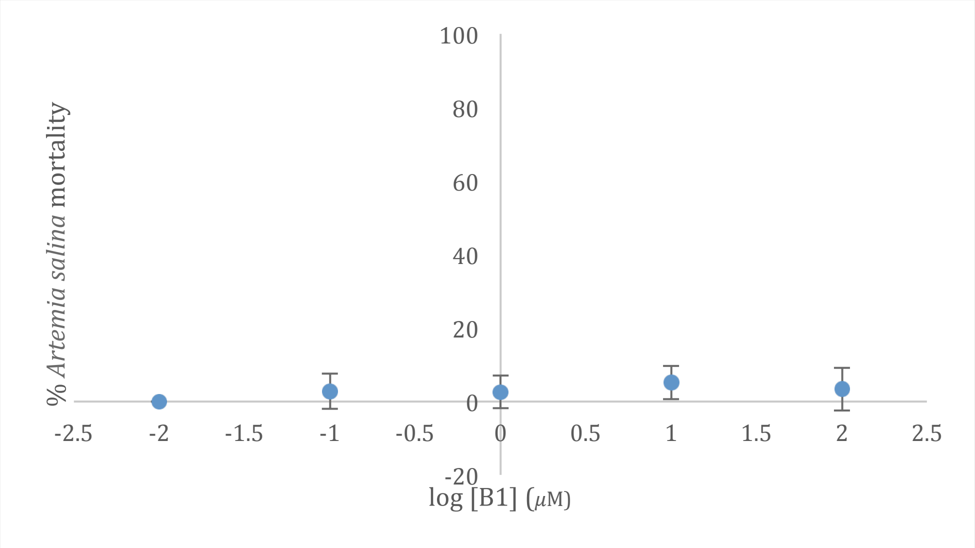

Figure S7. Mortality (%) of artemia salina *vs* log[**B1**] – dose–response curves.

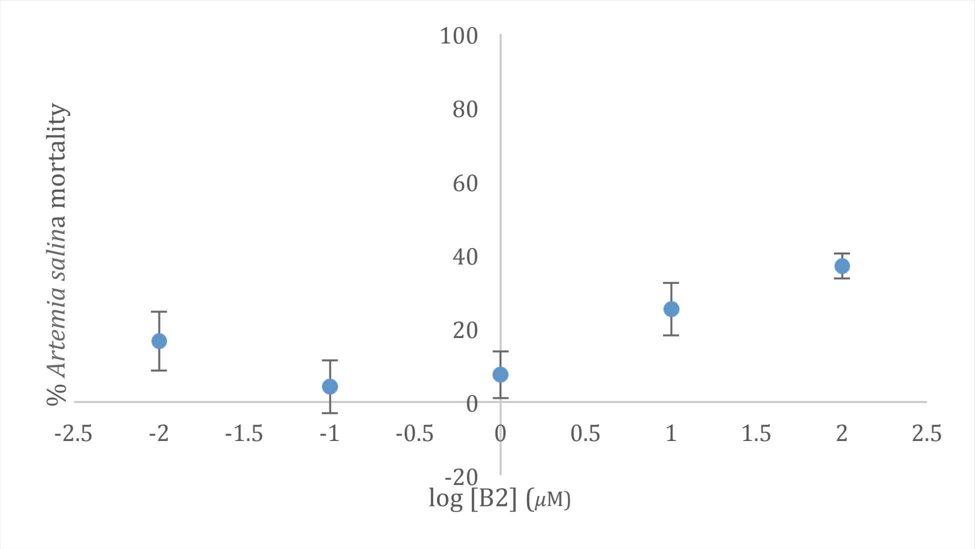

Figure S8. Mortality (%) of artemia salina *vs* log[**B2**] – dose–response curves.

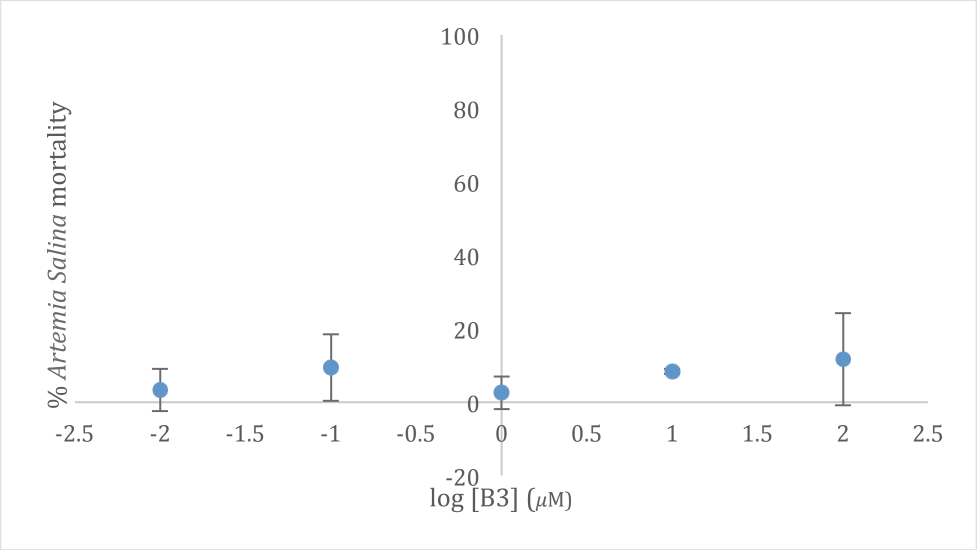

Figure S9. Mortality (%) of artemia salina *vs* log[**B3**] – dose–response curves.

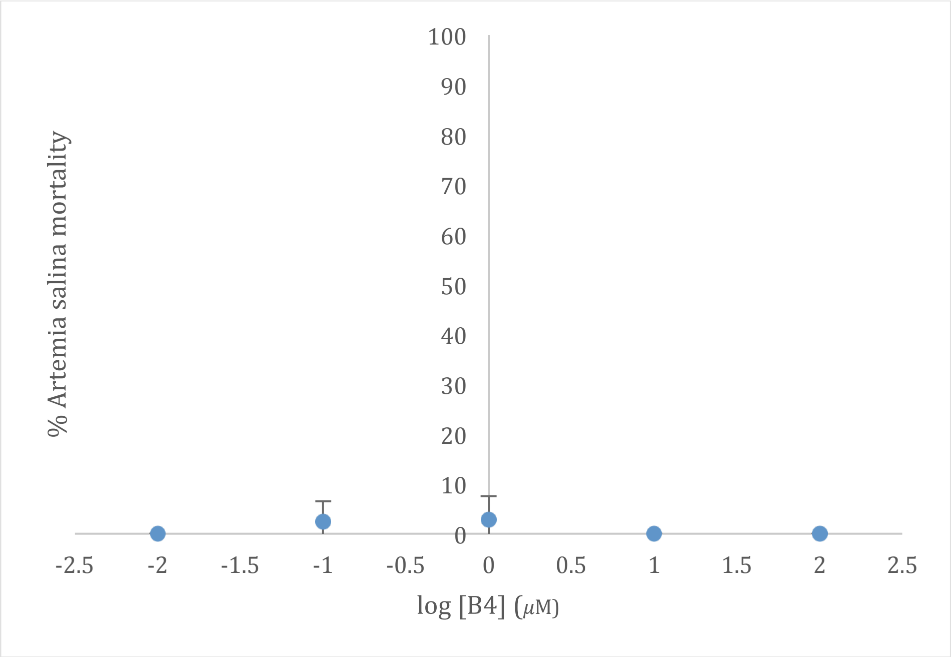

Figure S10. Mortality (%) of artemia salina *vs* log[B4] – dose–response curves.

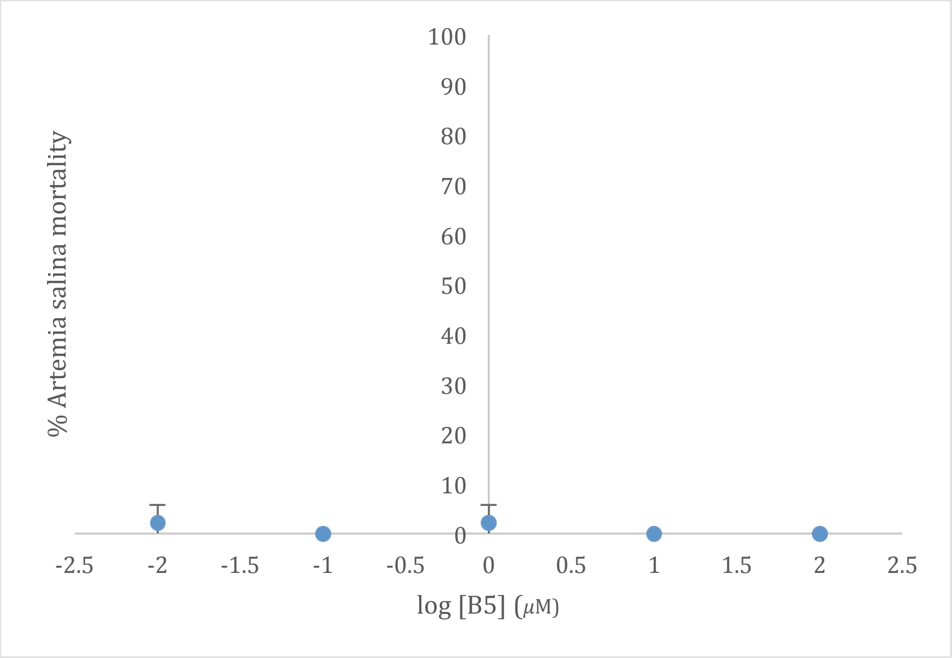

Figure S11. Mortality (%) of artemia salina *vs* log[**B5**] – dose–response curves.
